# A successful defense of the narrow-leafed lupin against anthracnose involves quick and orchestrated reprogramming of oxidation–reduction, photosynthesis and pathogenesis-related genes

**DOI:** 10.1038/s41598-022-12257-7

**Published:** 2022-05-17

**Authors:** Michał Książkiewicz, Sandra Rychel-Bielska, Piotr Plewiński, Wojciech Bielski, Maria Nuc, Bartosz Kozak, Paweł Krajewski, Małgorzata Jędryczka

**Affiliations:** 1 Department of Gene Structure and Function, Institute of Plant Genetics, Polish Academy of Sciences, Strzeszyńska 34, 60-479 Poznań, Poland; 2 Department of Genetics, Plant Breeding and Seed Production, Wroclaw University of Environmental and Life Sciences, Plac Grunwaldzki 24A, 50-363 Wrocław, Poland; 3 Department of Genetics and Plant Breeding, Faculty of Agronomy, Horticulture and Bioengineering, Poznan University of Life Sciences, Dojazd 11, 60-631 Poznań, Poland; 4 Department of Biometry and Bioinformatics, Institute of Plant Genetics, Polish Academy of Sciences, Strzeszyńska 34, 60-479 Poznań, Poland; 5 Department of Pathogen Genetics and Plant Resistance, Institute of Plant Genetics, Polish Academy of Sciences, Strzeszyńska 34, 60-479 Poznań, Poland

**Keywords:** Plant immunity, Plant molecular biology, Gene expression

## Abstract

Narrow-leafed lupin (NLL, *Lupinus angustifolius* L.) is a legume plant cultivated for grain production and soil improvement. Worldwide expansion of NLL as a crop attracted various pathogenic fungi, including *Colletotrichum lupini* causing a devastating disease, anthracnose. Two alleles conferring improved resistance, *Lanr1* and *AnMan*, were exploited in NLL breeding, however, underlying molecular mechanisms remained unknown. In this study, European NLL germplasm was screened with *Lanr1* and *AnMan* markers. Inoculation tests in controlled environment confirmed effectiveness of both resistance donors. Representative resistant and susceptible lines were subjected to differential gene expression profiling. Resistance to anthracnose was associated with overrepresentation of “GO:0006952 defense response”, “GO:0055114 oxidation–reduction process” and “GO:0015979 photosynthesis” gene ontology terms. Moreover, the *Lanr1* (83A:476) line revealed massive transcriptomic reprogramming quickly after inoculation, whereas other lines showed such a response delayed by about 42 h. Defense response was associated with upregulation of TIR-NBS, CC-NBS-LRR and NBS-LRR genes, pathogenesis-related 10 proteins, lipid transfer proteins, glucan endo-1,3-beta-glucosidases, glycine-rich cell wall proteins and genes from reactive oxygen species pathway. Early response of 83A:476, including orchestrated downregulation of photosynthesis-related genes, coincided with the successful defense during fungus biotrophic growth phase, indicating effector-triggered immunity. Mandelup response was delayed and resembled general horizontal resistance.

## Introduction

Narrow-leafed lupin (NLL, *Lupinus angustifolius* L.) is a high-protein grain legume species originating from the western part of the Mediterranean area^1,2^. It is currently cultivated as a grain crop for animal and human consumption. Moreover, it is appreciated in crop rotation system as a green manure due to nitrogen fixation by symbiotic diazotrophs and general improvement of the soil structure. NLL experienced rapid domestication process in the last century and is still remaining under high breeding pressure^3–12^. Widespread expansion of NLL cultivation has been followed by the succession of the pathogenic fungi, exploiting new agroecological niches and causing novel, yield damaging diseases. The most remarkable for lupin farmers and breeders was the appearance of anthracnose, caused by the pathogenic fungus, *Colletotrichum lupini* (Bondar) Nirenberg, Feiler & Hagedorn^13^. The earliest reports of the disease originate from Brazil and USA, where the typical symptoms were noted in 1912 and 1929, respectively. However, the causal agent was assigned about 30 years later^14^ as *Colletotrichum gloeosporioide*s (Penz.) Penz. & Sacc., teleomorph *Glomerella cingulata* (Stoneman) Spauld. & H. Schrenk,. Preliminary disease phenotyping performed in the middle of the twentieth century revealed some level of resistance in the NLL and yellow lupin (*L. luteus* L.) germplasm, but a high susceptibility of all tested white lupin (*L. albus* L.) accessions^15,16^. Moreover, it was revealed that the development of anthracnose correlates with the increase of rainfall (air humidity) and temperature (within the range 12–28 °C), resulting in the break of resistance at higher temperatures^17,18^. Indeed, under high humidity, time required for conidia germination and the onset of disease is fourfold shorter at 24 °C (4 h) than at 12 °C (16 h)^19^. Thus, the ongoing global warming contributes to the expansion of anthracnose. However, observation of this disease on white lupins in France (1982) and Ukraine (1983), that constituted a forerunner of forthcoming threat, was apparently overlooked by lupin industry at that time^20,21^. Several years later, this highly devastating disease appeared worldwide, affecting also key lupin producers, such as Australia, Poland, and Germany^22–24^. Following anthracnose outbreak in mid 1990s, large screening efforts have been undertaken, which resulted in identification of several resistance donors in NLL germplasm^19^. Resistance to anthracnose in NLL is controlled by two single dominant alleles that were found in different germplasm resources: *Lanr1* in the cultivars (cvs.) Tanjil and Wonga and *AnMan* in the cv. Mandelup^25,26^. These alleles were supplemented with molecular markers supporting selection of resistant germplasm in breeding programs^25–30^. Resistant 83A:476 breeding line carrying *Lanr1* allele was crossed with a susceptible P27255 wild accession to develop RIL population segregating for anthracnose resistance, which allowed assignment of the *Lanr1* locus to the chromosome NLL-11 by linkage mapping^31–33^. Alignment of markers from linkage maps flanking anthracnose resistance loci to the scaffolds of the NLL genome revealed localization of all three alleles in the same chromosome (NLL-11) but at different positions^29,34,35^. Nevertheless, due to small number of RILs and high genetic distance between markers and corresponding alleles^26,36^, it is not possible to draw reliable conclusions about the underlying genes. On the other hand, the use of reverse genetics in lupins is hampered by their very low regeneration potential, making genetic manipulation cumbersome^37^.

Development of domesticated germplasm carrying desired alleles in a homozygous state, such as 83A:476 (*Lanr1*) and Mandelup (*AnMan*), confronted with the presence of opposite allelic combinations in wild populations, opened the possibility of studying molecular mechanisms involved in anthracnose resistance by comparison of defense responses developed by particular genotypes. In this study, early transcriptomic response of NLL to inoculation with *C. lupini* was evaluated. First, the European NLL germplasm panel carrying 215 lines was screened with molecular markers tagging *Lanr1* and *AnMan* alleles. Then, the set of 50 NLL lines preselected by molecular markers was subjected to anthracnose disease phenotyping in controlled conditions. Based on these experiments, four lines differing in anthracnose resistance and *Lanr1*/*AnMan* allelic composition were chosen for differential gene expression profiling of defense response involving two complementary approaches: high-throughput RNA sequencing and real-time PCR quantification.

## Results

### Identification of candidate *Lanr1* and *AnMan* germplasm donors

Screening of NLL germplasm panel (N = 215) with *Lanr1* (Anseq3 and Anseq4) and *AnMan* (AnManM1) markers revealed that only one line (95726, Near Salamanca-b) amplified “resistant” alleles for all markers whereas the presence of “susceptible” scores for all markers was found in 158 (~ 73.5%) lines. 13 lines yielded “resistant” alleles for both *Lanr1* markers whereas 8 lines—a “resistant” allele for the *AnMan* marker (Supplementary Table S1). Two lines were heterozygous for the Anseq3 marker whereas one for the AnManM1 marker. 42 lines (19.5%) carried opposite phases of Anseq3 and Anseq4 alleles indicating high recombination frequency between these two loci. Anthracnose disease phenotyping in controlled environment (Supplementary Table S2) revealed the variability in resistance of the tested genotypes, reflected by differences in mean anthracnose disease severity scores, ranging from 1.8 (moderately resistant) to 6.9 (susceptible) as well as in differences in plant weight, ranging from 0.62 (susceptible) to 4.45 g (resistant). There was a significant correlation between values observed in two replications of the experiment (0.51 for disease severity scores, P = 0.00017 and 0.61 for plant weight, P < 0.0001) as well as between these two parameters (− 0.59 and − 0.77, P < 0.0001). The typical symptoms observed in susceptible plants included bending and twisting of stems resembling “shepherd’s crook” architecture, followed by occurrence of oval shaped lesions carrying orange/pink spore masses (Supplementary Fig. 1). Australian accessions carrying *Lanr1* (83A:476 and Tanjil) and *AnMan* (Mandelup) genes were found to be moderately resistant both by anthracnose scores (two-tailed mean comparison test P values 0.005, 0.017 and 0.0061) and mean weight measurements (P values 0.0031, 0.0331 and 0.0036). Some lines also carrying “resistant” *Lanr1* and/or *AnMan* alleles showed disease symptoms.

Interestingly, a few NLL lines lacking any “resistant” marker allele revealed a high level of anthracnose resistance (comparable or higher than for *Lanr1* or *AnMan* genotypes), such as Boregine (P value < 0.0001 for both parameters), Bojar (P value < 0.0001 for score and 0.001 for plant weight) and Population B-549/79b (P value < 0.0001 for score and non-significant for weight). Such a phenomenon indicated the hypothetical possibility of novel genetic sources of resistance, explaining the observed lack of correlation between marker genotypes and disease phenotypes (P values from ~ 0.42 to ~ 0.98). Thus, the Kolmogorov–Smirnov test showed that anthracnose resistance data were approximately normally distributed both for score (P values 0.25 and 0.11) and plant weight (P values 0.47 and 0.55), indicating and hypothetical involvement of more alleles than only *Lanr1* and *AnMan*.

### The link between *Lanr1* anthracnose resistance and rapid massive transcriptome reprogramming

Based on the results of anthracnose resistance screening, four lines were selected for transcriptomic profiling: 83A:476, Boregine, Mandelup and Population 22660. During the inoculation experiment for RNA sequencing, anthracnose resistance of these lines was assayed again, providing similar results to the previous test. The score values were as follows: Boregine (1.71 ± 1.39), 83A:476 (2.09 ± 1.38), Mandelup (3.82 ± 1.42) and Population 22660 (6.11 ± 1.29).

The Illumina NovaSeq 6000 protocol provided, on average, 40.5 M read pairs per sample (from 29.7 to 54.4 M) (Supplementary Table S3). The alignment rate in the reference sequence was from 75.5 to 88.6%. The mean correlation of the read count data within experimental variants between biological replications was from 0.812 to 0.997 (mean value 0.959). Out of the 35,170 genes analyzed, 2917 revealed no expression, and the other 4785 genes were expressed at negligible level (base mean < 5). Therefore, the number of genes considered to be expressed in the whole experiment (base mean ≥ 5) was 27,468 (78.1%) (Supplementary Table S4).

All NLL lines responded to *C. lupini* inoculation (strain Col-08) via transcriptome reprogramming since the first time point (Table 1); however, substantial differences between lines were observed. Thus, the resistant line 83A:476 (carrying *Lanr1* gene) revealed massive transcriptomic reprogramming at the first time point (6 hpi), highlighted by the 31–69 times higher number of up- and down-regulated genes than those observed in the other lines at this time point. Moreover, this peak was short-lived, as in the second time point (12 hpi) expression of just a few genes remained significantly altered. Interestingly, Boregine, which also showed a high level of resistance during inoculation tests, did not experienced such a massive transcription reprogramming during the course of the experiment. However, the number of differentially expressed genes (DEGs) in Boregine and 83A:476 at 12 hpi was similar. Both Mandelup and Population 22660 showed a DEG peak at the last time point (48 hpi), indicating relatively delayed defense response.

**Table 1:** Number of genes with significantly altered expression during response of narrow-leafed lupin lines to *Colletotrichum lupini* inoculation (strain Col-08, obtained in 1999 from the lupin field in Wierzenica, Poland).

Line	Response type	6 hpi^a^	12 hpi	24 hpi	48 hpi
83A:476	Repression	5264	2	60	5
(R^b^, *Lanr1*^c^)	Induction	2542	4	242	0
Boregine	Repression	49	9	65	22
(R, unknown)	Induction	62	7	63	251
Mandelup	Repression	94	85	14	1629
(MR, *AnMan*)	Induction	77	59	169	57
Population 22660	Repression	75	198	28	1796
(S, none)	Induction	178	23	116	591

^a^Hours post inoculation.

^b^Level of resistance to anthracnose (R, resistant; MR, moderately resistant; S, susceptible).

^c^Allele conferring resistance to anthracnose^25,26^.

**Figure 1:**
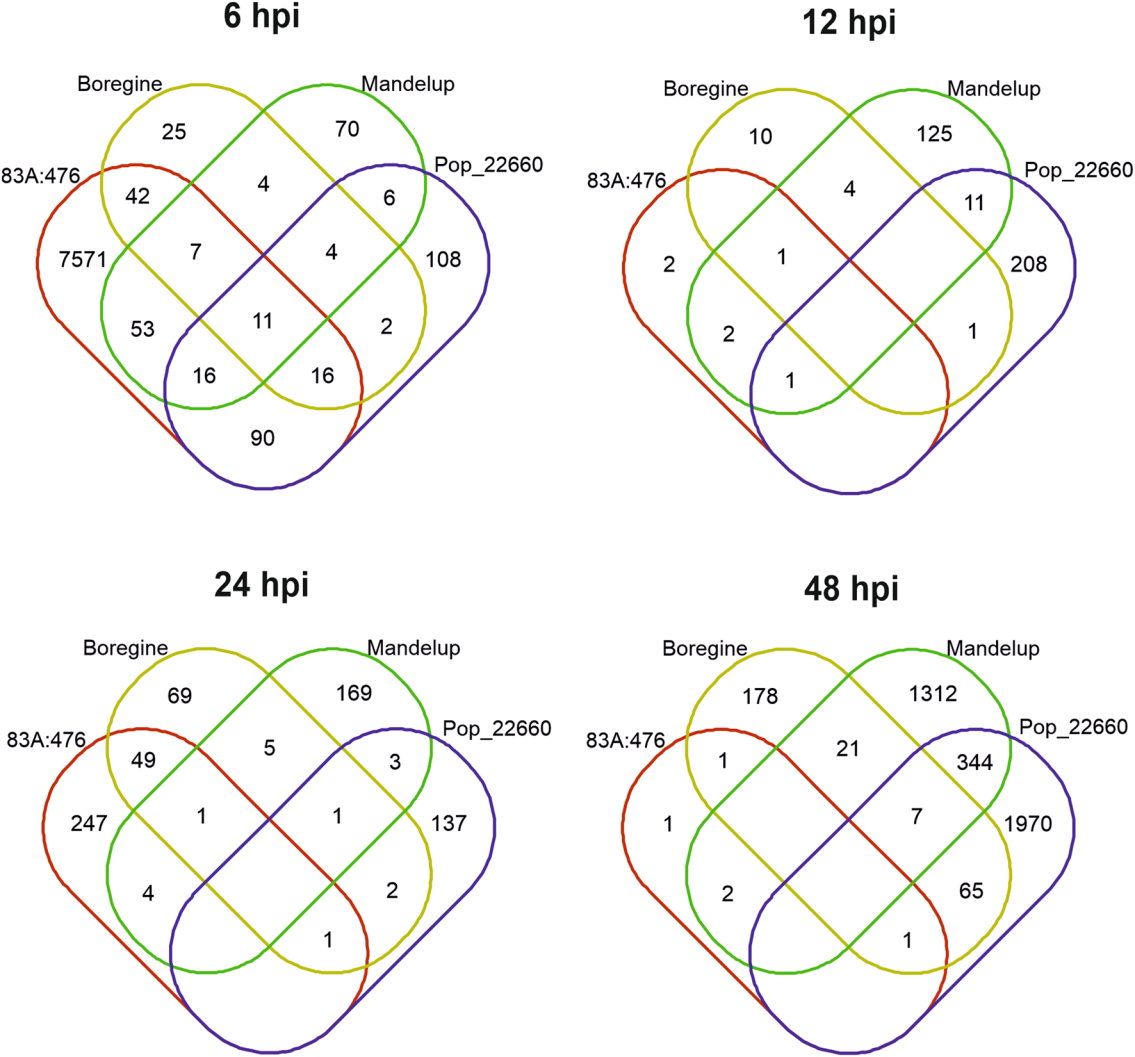
Venn diagrams showing the number of differentially expressed genes (DEGs) in narrow-leafed lupin (NLL) lines subjected to *Colletotrichum lupini* inoculation (strain Col-08, obtained in 1999 from the lupin field in Wierzenica, Poland). Analyzed NLL lines were as follows: 83A:476 (resistant, carrying the *Lanr1* allele), Boregine (resistant, unknown genetic background), Mandelup (moderately resistant, carrying *AnMan* allele) and Population 22660 (very susceptible). Abbreviation hpi stands for hours post inoculation. To simplify diagrams zero values were removed.

As 83A:476 experienced a massive transcriptome reprogramming in response to *C. lupini* at 6 hpi when compared to all other lines, ~ 91% of DEGs observed at this time point were specific to this line (Fig. 1). Nevertheless, there was some overlap in the early response between studied lines, as 68.5%, 50.9% and 52.6% of DEGs identified at 6 hpi in Boregine, Mandelup and Population 22660, respectively, overlapped with those revealed in 83A:476 at the same time point. However, these DEGs constituted only a small fraction (0.97–1.70%) of all DEGs revealed for 83A:476 at this time point. Moreover, 11 DEGs were coherent for all lines at this time point (Supplementary Tables S4-S6), including common components of plant defense response: a lipid transfer protein (*TanjilG_32225*), a glucan endo-1,3-beta-glucosidase (*TanjilG_23384*), two stress-induced proteins SAM22-like (*TanjilG_31528* and *TanjilG_31531*), a major latex protein (*TanjilG_32352*) and two glycine-rich cell wall structural proteins (*TanjilG_19701* and *TanjilG_19702*). There was also relatively high overlap in transcriptomic response between 83A:476 and Boregine at 24 hpi (shared 16–38% of DEGs) as well as between Mandelup and Population 22660 at 48 hpi (shared 14–20% of DEGs).

The set of genes upregulated at 6 hpi was analyzed for the presence of typical R gene domains^38^ (Supplementary Table S7). This survey revealed transcriptomic induction of classic disease resistance genes with NBS-LRR domains only in 83A:476. This set constituted one TIR-NBS-LRR gene (*TanjilG_05042*), five CC-NBS-LRR genes (*TanjilG_06165*, *TanjilG_06162*, *TanjilG_22773*, *TanjilG_22640* and *TanjilG_16162*) as well as four NBS-LRR genes (*TanjilG_06163*, *TanjilG_21020*, *TanjilG_27608* and *TanjilG_10386*). All these genes had typical domains localized in the conserved order. Besides NBS-LRR domain genes, some RLL kinases were upregulated at 6 hpi, namely one in Boregine (*TanjilG_19877*), two both in Mandelup (*TanjilG_07141* and *TanjilG_19877*) and Population 22660 (*TanjilG_09014* and *TanjilG_10361*), as well as twenty-seven in 83A:476.

### Defense response and oxidation–reduction processes are key components of *Lanr1* immune reaction

**Figure 2:**
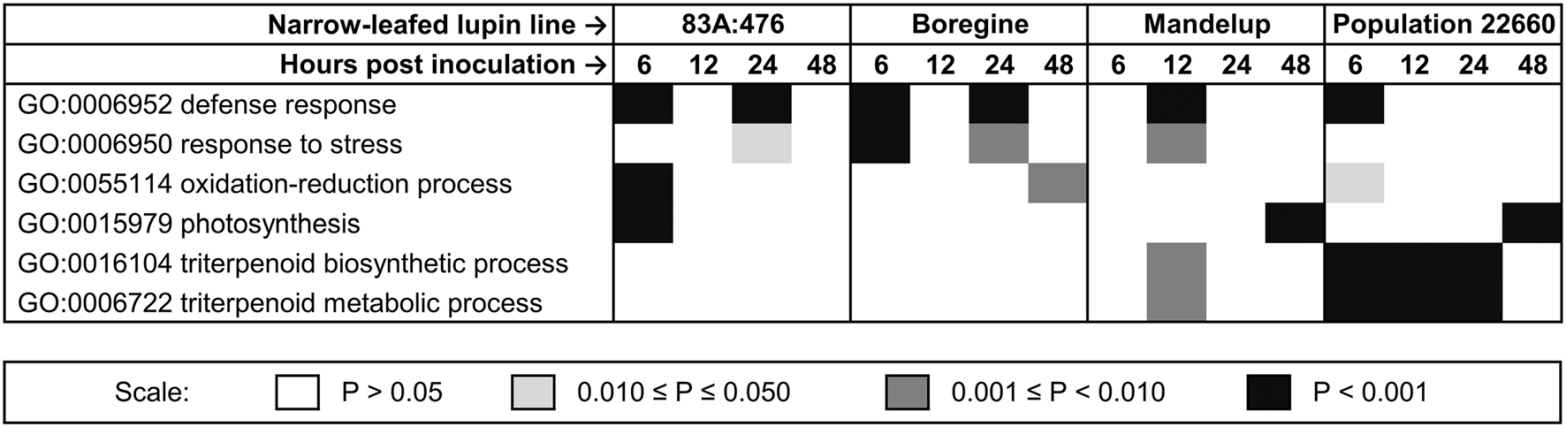
Selected biological process Gene Ontology terms significantly overrepresented in the annotation of differentially expressed genes during narrow-leafed lupin (NLL) transcriptomic response to inoculation with *Colletotrichum lupini* (strain Col-08, obtained in 1999 from the lupin field in Wierzenica, Poland). Analyzed NLL lines were as follows: 83A:476 (resistant, carrying homozygous *Lanr1* allele), Boregine (resistant, unknown genetic background), Mandelup (moderately resistant, carrying homozygous *AnMan* allele), and Population 22660 (susceptible).

Genes with expression significantly altered in response to *C. lupini* inoculation (strain Col-08) were subjected to a Gene Ontology (GO) enrichment analysis (Supplementary Table S8). The most frequently overrepresented biological process term was “GO:0006952 defense response” which appeared in 6 out of 16 (time × line) combinations with high significance (P value < 0.001) (Fig. 2). This term was overrepresented at two time points in 83A:476 and Boregine (6 hpi and 24 hpi) as well as at one time point in Mandelup and Population 22660 (12 hpi and 6 hpi, respectively). This is an expected outcome that highlights an antifungal response of resistant lines. Moreover, 83A:476 responded to *C. lupini* by quick induction of genes related with oxidative burst, represented by the term “GO:0055114 oxidation–reduction process” indicating specific defense response, whereas Boregine revealed significant activation of genes attributed to the term “GO:0006950 response to stress”. Population 22660 activated horizontal resistance reaction involving secondary metabolites, highlighted by overrepresentation of terms “GO:0016104 triterpenoid biosynthetic process” and “GO:0006722 triterpenoid metabolic process” (both terms are attributed to the same set of genes). Taking into consideration the results of GO term enrichment analysis, resistance response of Mandelup intermediated between Boregine and Population 22660. Moreover, early response of 83A:476 (6 hpi) and delayed responses of Mandelup and Population 22660 included GO:0015979 term “photosynthesis” and other related biological processes.

As this study was aimed at identification of genes contributing to the development of anthracnose resistance, an analysis of genes assigned to GO terms “GO:0006952 defense response” and “GO:0055114 oxidation–reduction process” was performed, with threshold of base mean count ≥ 30 and statistically significant log2(fold-change) value for at least one line × time point combination. The number of genes fulfilling these criteria was 65 for the GO:0006952 and 524 for the GO:0055114.

**Figure 3:**
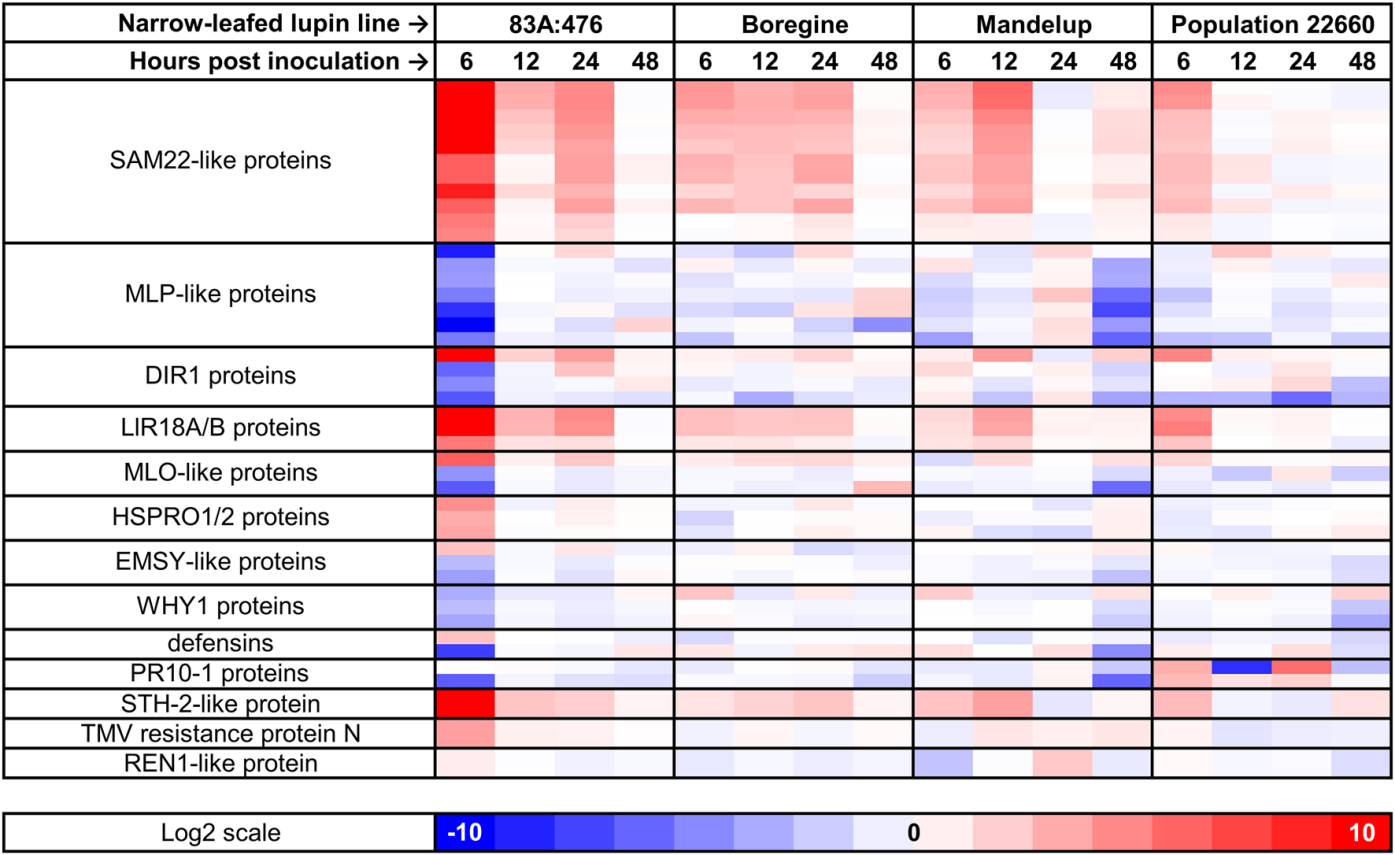
Expression patterns revealed for major components of biological process term “GO:0006952 defense response” and candidate genes for *Lanr1* and *AnMan* alleles. Log2 scale represents the log2(fold-change) values between inoculated (*Colletotrichum lupini*, strain Col-08, obtained in 1999 from the lupin field in Wierzenica, Poland) and control (mock inoculation) plants at the same time point. Analyzed narrow-leafed lupin lines were as follows: 83A:476 (resistant, carrying homozygous *Lanr1* allele), Boregine (resistant, unknown genetic background), Mandelup (moderately resistant, carrying homozygous *AnMan* allele), and Population 22660 (susceptible).

83A:476 revealed two peaks of DEGs annotated by the GO:0006952 term, the first at 6 hpi (64 genes, up- and down-regulated) and the second at 24 hpi (15 genes, only up-regulated). Boregine also revealed GO:0006952 peaks at the same time points, however with lower number of DEGs (11 and 8) and domination of up-regulation. Mandelup showed two GO:0006952 peaks at 12 and 48 hpi, both carrying 12 genes (the first with up-regulated and the second with down-regulated genes only), whereas Population 22660 had one major peak at 6 hpi (13 genes) with the prevalence of upregulation. It should be noted that 96.4% of GO:0006952 DEGs in these peaks had the same the type of response (up- or down-regulation), indicating significant overlap in defense responses despite differences in the number of genes involved. The largest groups of sequences attributed to GO:0006952 term encoded stress-induced starvation-associated message 22 (SAM22-like) proteins, belonging to the pathogenesis-related class 10 protein (PR-10) clade, and major latex protein-like (MLP-like) proteins (Fig. 3). These two groups differed by expression patterns and direction of the response. Genes encoding SAM22-like proteins revealed coherent and significant induction at the early time points (6 or 12 hpi) tending to non-responsiveness at the end of experiment (48 hpi), whereas MLP-like protein genes revealed orchestrated downregulation at 6 hpi in 83A:476 and at 48 hpi in Mandelup, and non-responsiveness at almost all remaining data points. Moreover, differences in expression profiles of SAM22-like protein genes followed observed variability in anthracnose resistance, as more resistant lines had more time points with significant induction of these genes than the more susceptible ones. The other PR-10 genes, LlR18A/B-like, revealed expression patterns very similar to those of SAM22-like protein genes.

Additionally, RNA-seq expression profiles of candidate genes for *Lanr1* (*TanjilG_05042*) and *AnMan* (*TanjilG_12861*) alleles were evaluated (Fig. 3). *TanjilG_05042* gene revealed significant response (upregulation) only in 83A:476 at the first time point (6 hpi), whereas *TanjilG_12861* was significantly responsive only in Mandelup at two time points: 6 hpi (downregulation) and 24 hpi (upregulation).

**Figure 4:**
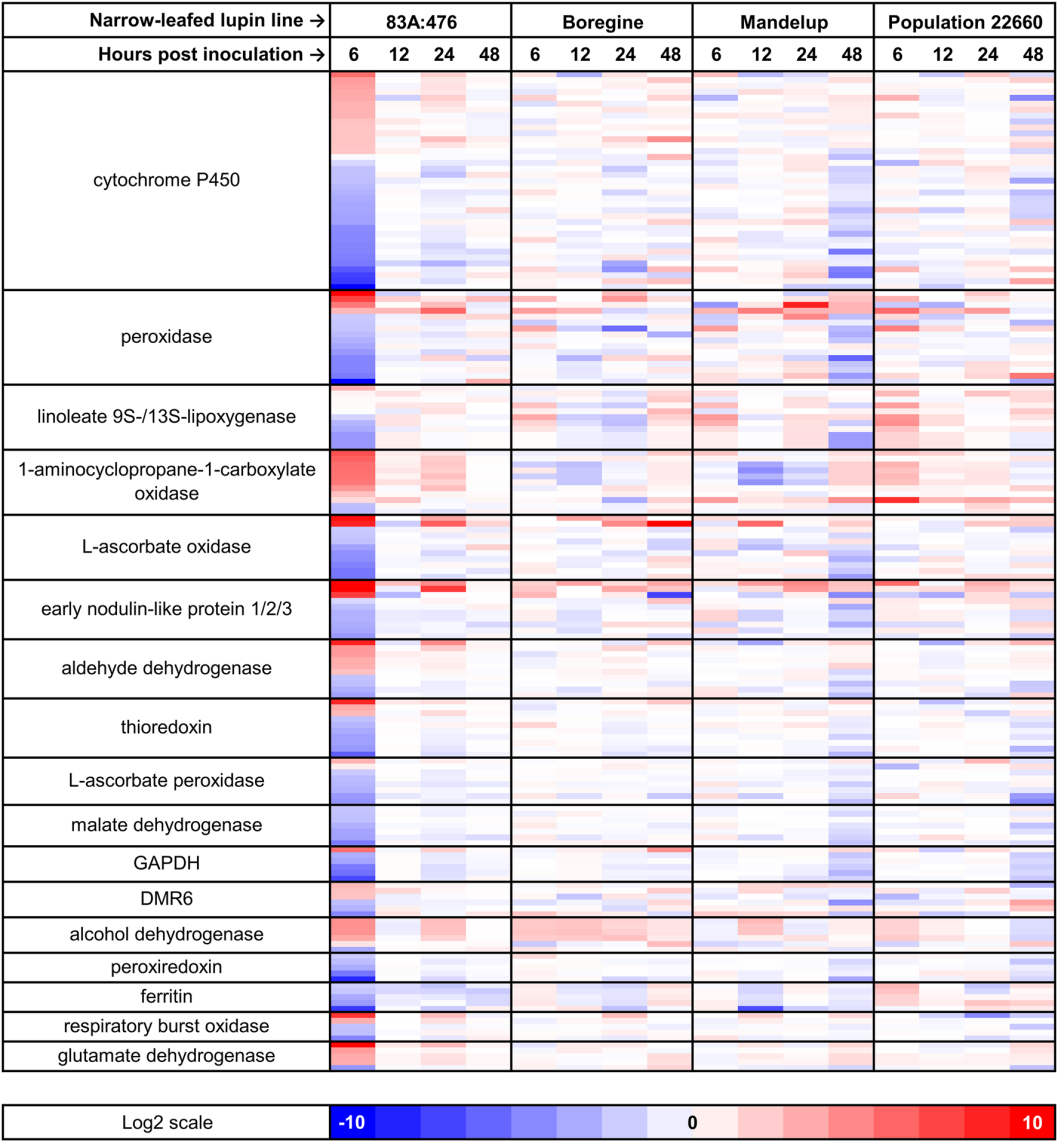
Expression patterns revealed for major components of biological process term “GO:0055114 oxidation–reduction process”. Log2 scale represents the log2(fold-change) values between inoculated (*Colletotrichum lupini*, strain Col-08, obtained in 1999 from the lupin field in Wierzenica, Poland) and control (mock inoculation) plants at the same time point. Analyzed narrow-leafed lupin lines were as follows: 83A:476 (resistant, carrying homozygous *Lanr1* allele), Boregine (resistant, unknown genetic background), Mandelup (moderately resistant, carrying homozygous *AnMan* allele), and Population 22660 (susceptible).

The most numerous genes overrepresented in GO:0055114 term “oxidation–reduction process” were those encoding cytochrome P450 proteins and peroxidases (Fig. 4). The maximum or minimum log2(fold-change) values between inoculated and control plants were usually (for 86.6% of genes) observed for samples isolated from 83A:476 at 6 hpi, highlighting high responsiveness of this genotype to inoculation. 83A:476 revealed the highest number of significant GO:0055114 DEGs at 6 hpi (503 genes), whereas the remaining lines—at 48 hpi (Boregine, 31 genes; Mandelup, 85 genes; and Population 22660, 78 genes). In the majority of GO:0055114 gene families both types of response (activation and repression) to inoculation were observed. Interestingly, as many as 97.6% of DEGs identified in Mandelup at 48 hpi for the GO:0055114 term were also found, with the same direction of response (up/down-regulation), in 83A:476 at 6 hpi. Such an observation indicates that despite the significantly lower scale (i.e., number of redox genes with altered expression, 85 vs 503), the pattern of delayed transcriptomic response of Mandelup to the anthracnose was similar to the early response of 83A:476. In Boregine and Population 22660 this convergence was lower, accounting for 51.6% and 75.6% DEGs, respectively.

**Figure 5:**
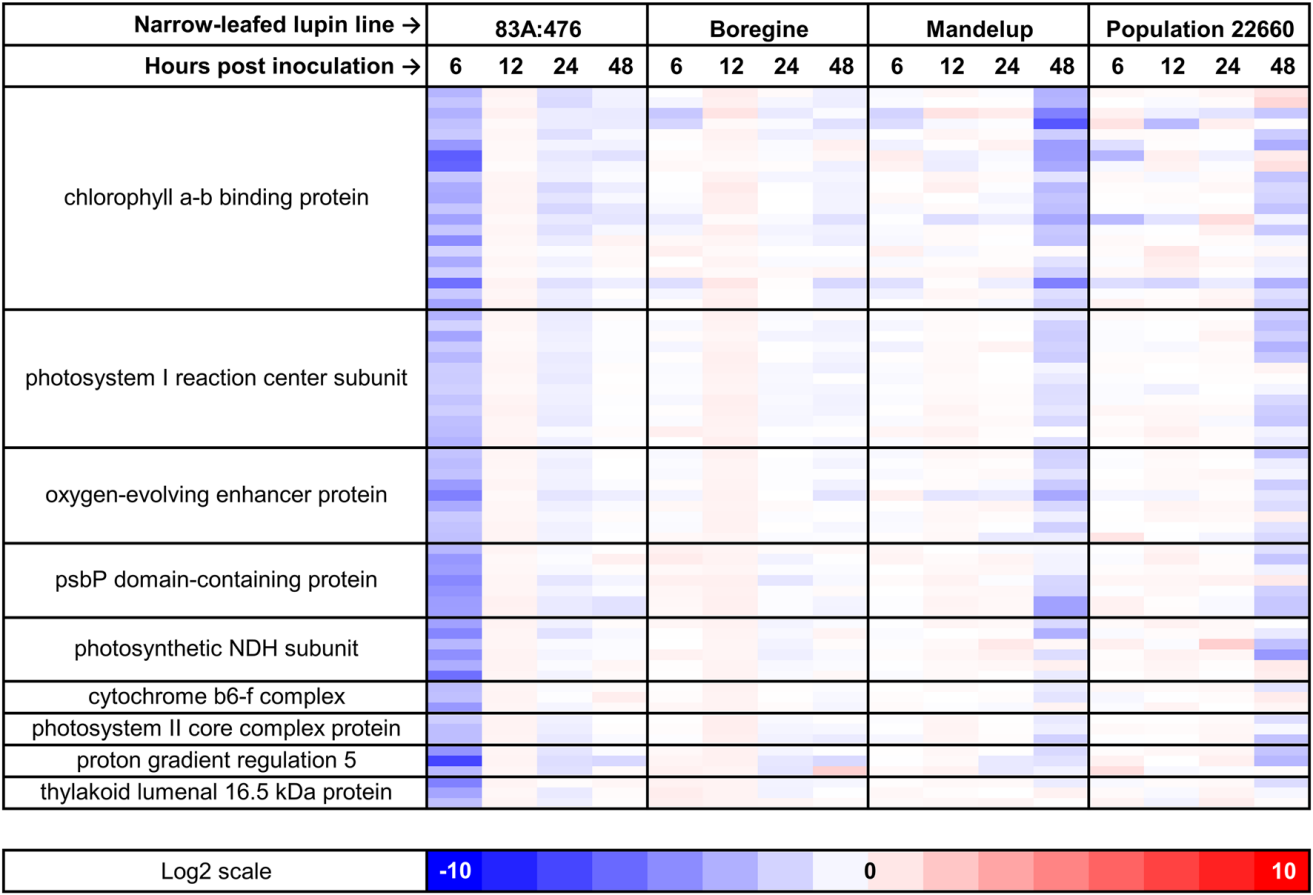
Expression patterns revealed for major components of biological process term “GO:0015979 photosynthesis”. Log2 scale represents the log2(fold-change) values between inoculated (*Colletotrichum lupini*, strain Col-08, obtained in 1999 from the lupin field in Wierzenica, Poland) and control (mock inoculation) plants at the same time point. Analyzed narrow-leafed lupin lines were as follows: 83A:476 (resistant, carrying homozygous *Lanr1* allele), Boregine (resistant, unknown genetic background), Mandelup (moderately resistant, carrying homozygous *AnMan* allele), and Population 22660 (susceptible).

Transcriptomic response of 83A:476 to inoculation with *C. lupini* (strain Col-08) included also orchestrated downregulation of genes attributed to the GO:0015979 term “photosynthesis” and other related biological processes (Fig. 5). The set of GO:0015979 DEGs encompassed 105 genes significantly repressed at 6 hpi in 83A:476. Out of this subset, 37 genes were also downregulated in Mandelup at 48 hpi and 35 in Population 22660 at the same time point, including 19 DEGs common for both genotypes. There was no DEG attributed to the GO:0015979 term which was significantly upregulated at any of the (line × time point) combinations.

### Results of quantitative PCR

Based on the results of differential expression analysis and hypothetical involvement in defense response against pathogenic fungi, the set of 7 genes was selected for quantification of expression profiles by real-time PCR (Supplementary Table S9).

**Figure 6:**
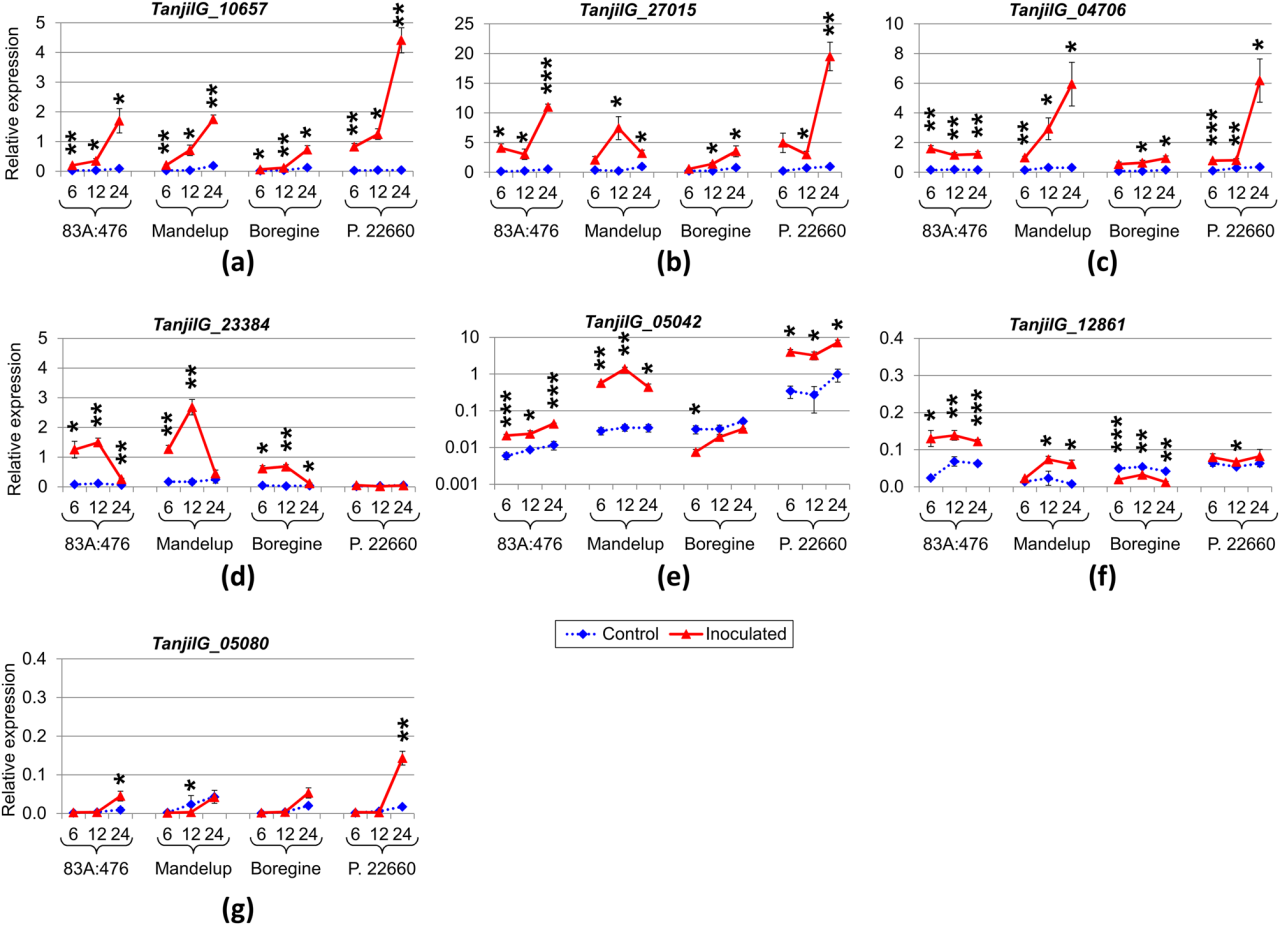
Expression profiles of selected genes (**a**–**g**) revealed by quantitative PCR. The numbers 6, 12, and 24 stand for hours post inoculation. *LanDExH7* and *LanTUB6* genes were used for the normalization, and *LanTUB6* for the inter-run calibration. The error bars indicate the standard deviation based on three biological replicates, each representing a mean of three technical replicates. The statistical significance of differences in the expression levels between the inoculated (*Colletotrichum lupini*, strain Col-08, obtained in 1999 from the lupin field in Wierzenica, Poland) and control (mock-inoculated) plants are marked above data points (*P value < 0.05, **P value ≤ 0.01, ***P value ≤ 0.001). Analyzed NLL lines were as follows: 83A:476 (resistant, carrying homozygous *Lanr1* allele), Mandelup (moderately resistant, carrying homozygous *AnMan* allele), Boregine (resistant, unknown genetic background), and Population 22660 (susceptible).

A hypothetical protein gene *TanjilG_10657* was revealed to be significantly induced in all studied lines and time points if compared to control (mock-inoculated) plants (Supplementary Tables S10, S11). Moreover, expression profile of *TanjilG_10657* revealed increasing trend during the progress of experiment in all lines. Population 22660 showed the highest responsiveness of *TanjilG_10657* to inoculation, manifested by up to 114-fold upregulation and the highest relative expression level (4.4 ± 0.4) at 24 hpi (Fig. 6a). A PR10 protein LlR18A gene *TanjilG_27015* also revealed upregulation in all lines and time points with statistical significance in the majority of data points (Fig. 6b). Similarly to *TanjilG_10657*, the highest relative expression level of *TanjilG_27015* was observed in inoculated Population 22660 at 24 hpi (19.5 ± 2.4). An acidic endochitinase gene *TanjilG_04706* was significantly upregulated in all lines and time points except Boregine at 6 hpi (Fig. 6c). It was highly induced at the first time point (6 hpi) in 83A:476 (10.5-fold) as well as moderately upregulated in the remaining lines (6.6–7.5-fold). During the course of experiment, *TanjilG_04706* expression remained at similar levels 83A:476 and Boregine, whereas in Mandelup and Population 22660 it significantly increased, reaching relatively high values (5.9 ± 1.5 and 6.2 ± 1.5, respectively). A glucan endo-1,3-beta-glucosidase-like gene *TanjilG_23384* revealed high upregulation in the first two time points (6 and 12 hpi) in all lines except Population 22660 (Fig. 6d). The highest relative expression levels of *TanjilG_23384* were observed in the second time point (12 hpi) in Mandelup (2.7 ± 0.3) and 83A:476 (1.5 ± 0.1). At 24 hpi expression of *TanjilG_23384* was relatively low in all studied lines (from 0.04 ± 0.009 to 0.44 ± 0.12).

A candidate gene for the *Lanr1* locus, *TanjilG_05042*, revealed expression pattern that significantly differed from the profile derived from the RNA-seq study (Fig. 6e). Significant upregulation of this gene was observed in Mandelup and Population 22660 (up to 39.7-fold and 11.7-fold, respectively), resulting in relatively high expression levels (up to 1.4 ± 0.14 and 7.2 ± 1.3, respectively). 83A:476 revealed also some upregulation of *TanjilG_05042* gene (up to 3.8-fold), however, the achieved relative expression level (0.044 ± 0.002) was more than 30 times lower than those observed in Mandelup and Population 22660. Moreover, it was the only gene from those analyzed by quantitative PCR that showed significant differences in expression levels between genotypes in mock-inoculated (control) variant, reaching 58-fold difference between Population 22660 and 83A:476 and ~ 11-fold difference between Population 22660 and Boregine or Mandelup lines.

A candidate gene for the *AnMan* locus, *TanjilG_12861*, was upregulated in response to inoculation in 83A:476 and Mandelup, neutral in Population 22660 and downregulated in Boregine (Fig. 6f). Relative expression level of *TanjilG_12861* gene was the highest in inoculated 83A:476 (0.14 ± 0.01). The 17.4 kDa class I heat shock protein HSP17.4 gene, *TanjilG_05080*, revealed low relative expression levels in all studied lines and time points (Fig. 6g). The highest value was observed at 24 hpi in Population 22660 (0.14 ± 0.02, eightfold increase in response to inoculation).

**Figure 7:**
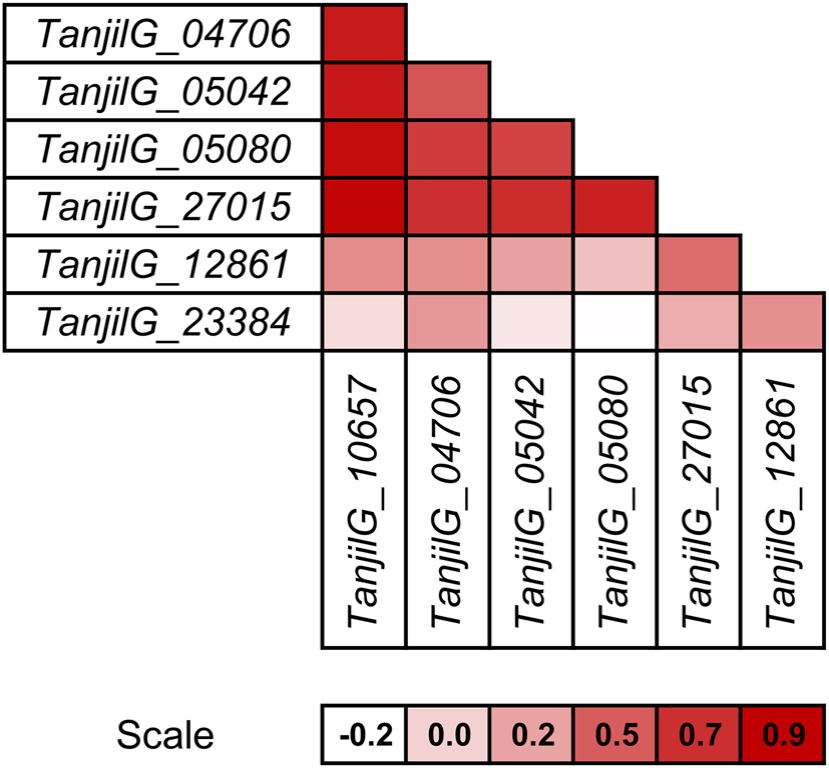
Correlations between gene expression profiles revealed using quantitative PCR. Analyzed narrow-leafed lupin lines were as follows: 83A:476 (resistant, carrying homozygous *Lanr1* allele), Mandelup (moderately resistant, carrying homozygous *AnMan* allele), Boregine (resistant, unknown genetic background), and Population 22660 (susceptible). Calculations were performed for three time points (6, 12, and 24 stand for hours post inoculation) including results obtained for inoculated (*Colletotrichum lupini*, strain Col-08, obtained in 1999 from the lupin field in Wierzenica, Poland) and control (mock-inoculated) plants. Scale shows Pearson correlation coefficient values.

Comparison of gene expression profiles (Fig. 7) revealed high correlation between *TanjilG_10657* and four other genes: *TanjilG_27015* (r = 0.89), *TanjilG_05080* (r = 0.85), *TanjilG_05042* (r = 0.80) and *TanjilG_04706* (r = 0.79). Such a result may indicate joint co-regulation of these genes during defense response. Genes *TanjilG_12861* and *TanjilG_23384* revealed distinct expression profiles, highlighted by low Pearson correlation coefficient values as compared to other genes (from 0.08 to 0.43 and from -0.19 to 0.28, respectively).

### Weighted gene co-expression network analysis (WGCNA)

**Figure 8:**
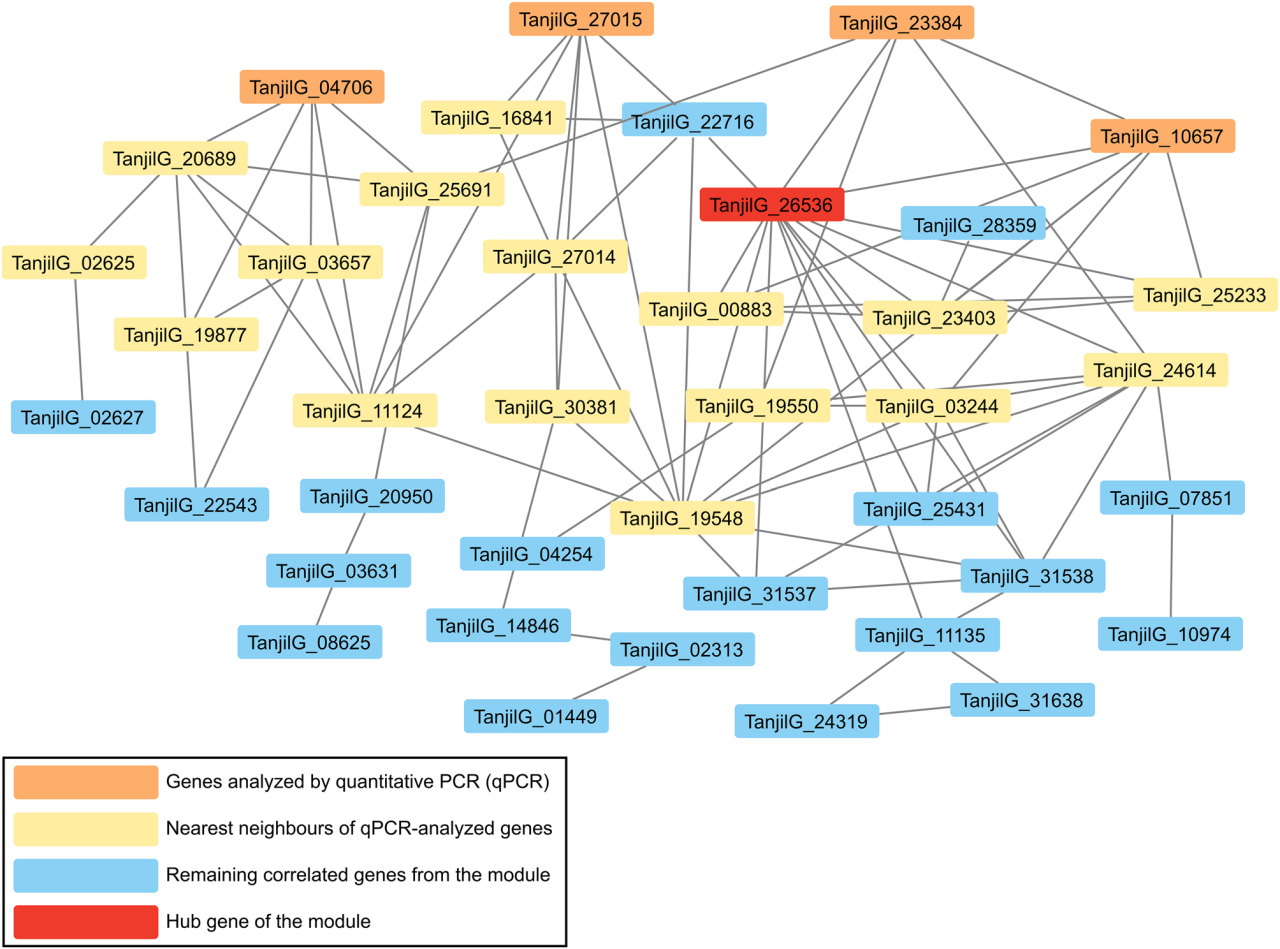
Weighted gene co-expression network analysis for a module with overrepresented biological process term “GO:0006952 defense response”. The connections are simplified to highlight four genes analyzed by quantitative PCR (*TanjilG_04706*, *TanjilG_23384*, *TanjilG_10657* and *TanjilG_27015*).

**Figure 9:**
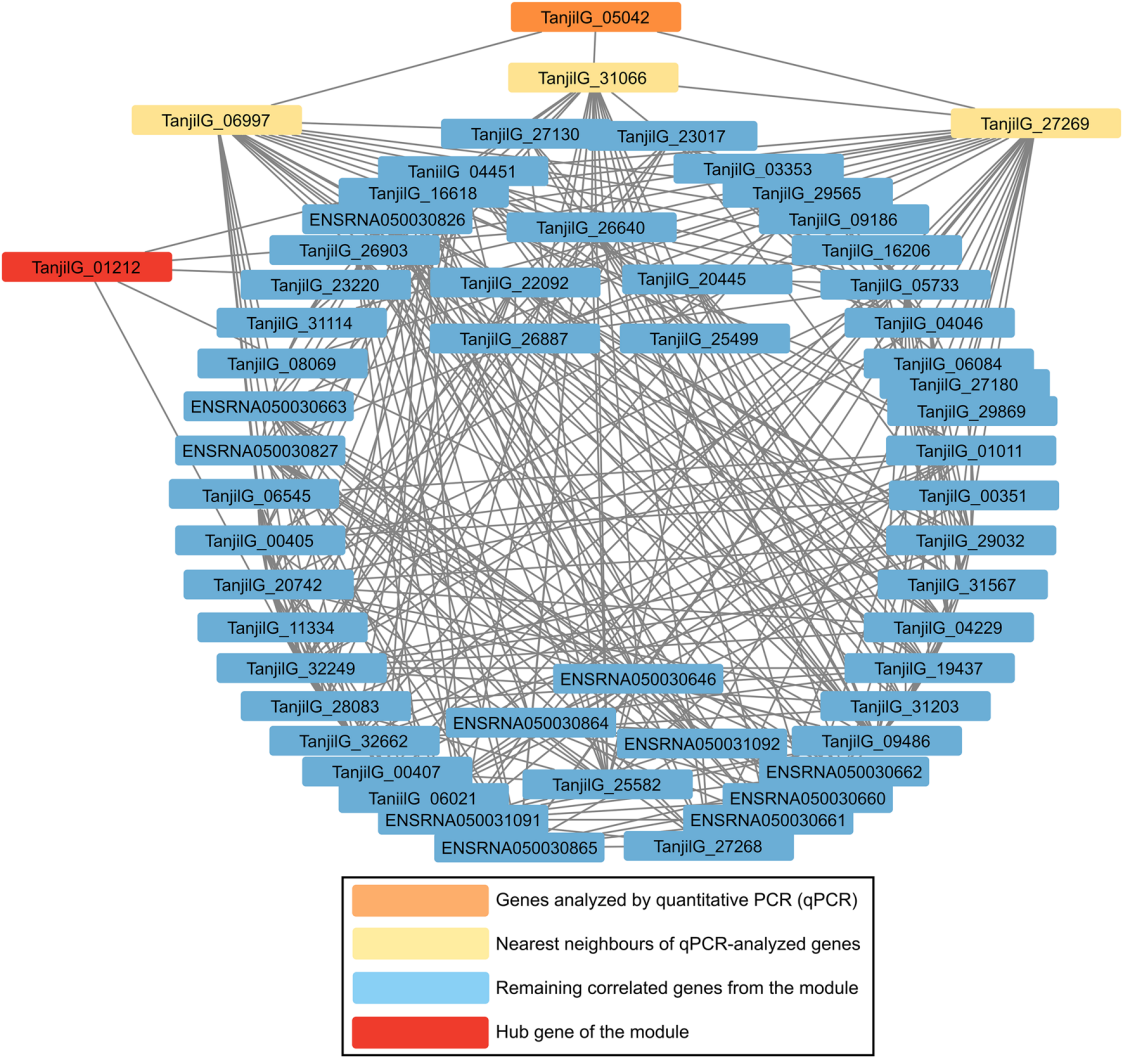
Weighted gene co-expression network analysis for a module with overrepresented biological process term “GO:0006355: regulation of transcription, DNA-templated” and carrying a candidate *Lanr1* anthracnose resistance gene *TanjilG_05042*. The connections are simplified to highlight *TanjilG_05042* gene and a hub gene *TanjilG_01212*.

WGCNA was performed for 9981 DEGs identified in comparisons between inoculated and control plants, based on data obtained at 6 hpi to focus on early defense response (Supplementary Table S12). Twenty two modules (clusters) of genes with correlated (positively or negatively) expression profiles over genotypes and experimental variants were found. On average, gene expression levels were descending in order 83A:476 > Mandelup > Boregine > Population 22660 (in both variants, however, this trend was stronger in control plants). Inoculation resulted in upregulation of gene expression, especially in modules 18, 19, 14, 6 and 1 (sorted by descending order of effects), downregulation (e.g., modules 9 and 20) or had neutral effect (e.g. modules 11, 22, 8, and 13). GO term enrichment analysis (Supplementary Table S13) revealed “GO:0006952 defense response” for the most inoculation-upregulated module (18) that included genes analyzed by quantitative PCR (*TanjilG_04706*, *TanjilG_23384*, *TanjilG_10657* and *TanjilG_27015*), and numerous GO terms related with photosynthesis for the most inoculation-downregulated module (9). The hub of the module 18 (Fig. 8) was identified as *TanjilG_26536* gene encoding PR-10 class protein LlR18B, whereas the hub of the module 9—as *TanjilG_28955* gene encoding photosystem II PsbQ protein. A candidate *Lanr1* anthracnose resistance gene, *TanjilG_05042*, was found in the module 22 (Fig. 9), associated with terms “GO:0044260 cellular macromolecule metabolic process” and “GO:0006355 regulation of transcription, DNA-templated”, carrying a hub at *TanjilG_01212* gene encoding a heat stress transcription factor A-4a (*HSFA4a*).

## Discussion

### NLL alleles conferring anthracnose resistance

The screening of Australian collection for anthracnose resistance revealed that the most of the early released cultivars were susceptible; Kalya, Coromup and Mandelup were described as moderately resistant, whereas Wonga, Tanjil and 83A:476 as highly resistant^26,27,31^. Disease nurseries performed on the progeny descending from crosses between particular accessions and cultivars demonstrated that Wonga, Tanjil and 83A:476 possess the same resistance allele, named *Lanr1*, Coromup and Mandelup have the other allele, named *AnMan*^10,26,39^, whereas Kalya confers another allele, *Lanr2*. Screening for anthracnose resistance in Germany resulted in identification of resistant line Bo7212 carrying different candidate allele than *Lanr1*, named *LanrBo*^36^.

Our study revealed very low frequency of the *Lanr1* allele in tested germplasm panel (about 6%). This observation converges with the result of Eastern European germplasm screening with Anseq3 and Anseq4 markers, which evidenced the presence of *Lanr1* allele only in two Belarusian lines^40^. It indicates that *Lanr1* allele has not been widely exploited by local breeding programs, contrary to Australia, where it was one of the key alleles targeted by marker-assisted selection^30^. It might be related with the lower level of resistance provided by *Lanr1* allele in European field conditions as compared to Australian reports^41^. Moreover, anthracnose surveys in high rainfall sites in Australia revealed that the resistance response driven by *Lanr1* allele might be inefficient in weather conditions favouring pathogen growth and promoting its faster development^19,42^. Indeed, in the present study some anthracnose disease symptoms were also observed in genotypes carrying *Lanr1* alleles, indicating possible breakdown of resistance in conditions optimal for *C. lupini* development. Moreover, there is also a chance of false-positive interpretation of the presence of Anseq3 and Anseq4 markers, which are located approximately 1 cM away from the *Lanr1* locus^28,30,43^.

### The onset of anthracnose resistance is associated with rapid molecular response to inoculation

Our study revealed that 83A:476 carrying *Lanr1* allele responded to inoculation with *C. lupini* by massive transcriptome reprogramming at the first analyzed time point (6 hpi), whereas transcriptomic response was observed in Mandelup carrying *AnMan* allele was considerably delayed (from 24 to 48 hpi). This time shift in defense response was associated with differences in disease symptoms, underlining the significance of early pathogen recognition for the successful resistance response. To infect plant tissues, *Colletotrichum* spores must complete several developmental stages on the host surface, including germination, cell division and formation of an appressorium. An appressorium is an infection structure that adheres to the host surface and facilitates penetration of host tissues^44^. Thus, *C. gloeosporioides* spores in pea extract revealed first nuclear division after just 75–90 min of incubation, germ tube formation after 90–120 min and appressorium appearance after 4 h^45^. Mango *C. gloeosporioides* revealed above 40% of conidia germination at 3 h of incubation and ~ 20% of appressorium formation at 4 h. A *C. gloeosporioides* virulence-related *CAP20* gene showed transcriptional activity in appressoria-forming conidia after 3.5 h of incubation in avocado surface wax and high concentration of CAP20 protein at 4 h^46^. Similarly, activity of melanin biosynthetic genes in *C. trifolii* was induced at 2 h incubation time, followed by appresorium formation one hour later^47^. Studies on leaf tissues revealed the presence of the first appressoria at 8 hpi for strawberry inoculated with *C. acutatum* and at 4 hpi for tomato inoculated with *C. coccodes*^48,49^. Thus, deployment of defense response in the resistant line 83A:476 highly corresponds with the timeline of *Colletotrichum* spp. infection process. Quick defense response of 83A:476 suggests an involvement of plant resistance gene(s) and effector-triggered immunity (ETI) in this line, whereas delayed response in Mandelup supports the hypothesis on microbe-associated molecular pattern-triggered immunity (MTI)^50^. Revealed partial overlap in upregulated or downregulated genes between early response of 83A:476 and delayed response of Mandelup also support this concept, because ETI is generally considered as an accelerated and amplified MTI response eventually leading to the programmed cell death at the infection site known as hypersensitive response^51,52^.

### Early responsiveness of pathogenesis-related protein PR-10 genes to anthracnose

A majority of genes attributed to the overrepresented Gene Ontology term GO:0006952 “defense response” were 11 homologs of stress-induced protein starvation-associated message 22 (SAM22-like) and 7 homologs of major latex protein-like (MLP-like) showing sequence similarity to MLP-like 31, 34, 43 and 423 proteins. SAM22-like genes revealed significant upregulation which lasted longer in lines that showed increased level of resistance to anthracnose (83A:476 and Boregine). MLP-like genes were downregulated, however, only in lines carrying candidate resistance alleles (83A:476/*Lanr1* at 6 hpi and Mandelup/*AnMan* at 24 hpi). It should be noted that all identified SAM22-like homologs originated from one gene cluster spanning ~ 105 kbp whereas MLP-like genes originated from different regions of the genome. Such an orchestrated activation of SAM22-like genes was also identified in our previous study on the NLL resistance to stem inoculation with *Diaporthe toxica*^53^, that suggests their involvement in the horizontal component of defense response. This conclusion is also supported by the report on the positive responsiveness of SAM22-like genes to wounding or treatments with salicylic acid, fungal elicitor or hydrogen peroxide^54^.

MLP-like genes were evidenced to be responsive to various abiotic and biotic stresses, including infections with bacteria, viruses and pathogenic fungi in numerous plant species^55^. Direction of the response varied between particular plant–pathogen interactions, from high upregulation (i.e. during cotton infection with fungus *Verticillium dahliae*) to significant downregulation (i.e. after apple infection with fungus *Alternaria alternata*)^56,57^. Significant downregulation of an MLP-like 423 gene was observed during avocado defense response to *Fusarium kuroshium* infection as well as during apple infections with *Botryosphaeria berengeriana* f. sp. *piricola* and *Alternaria alternata* apple pathotype^58,59^. Moreover, apple calli overexpressing an MLP-like 423 gene had lower expression of resistance-related genes, and were more sensitive to fungal infections^59^. MLP-like 423 gene was also downregulated in a resistant common bean accession after *Fusarium oxysporum* f. sp. *phaseoli* infection^60^.

Other members of PR-10 family quarried by our RNA-seq study were *LlR18A* and *LlR18B* genes which responded by upregulation, and lipid transfer protein *DIR1* genes which were upregulated (1 gene) or downregulated (3 genes). Moreover, WGCNA highlighted the *LlR18B* gene as a hub in the module that was highly responsive to inoculation and carried several defense response genes. The *LlR18A* and *LlR18B* genes were induced in yellow lupin leaves in response to pathogenic bacteria as well as in the NLL stems after inoculation with *D. toxica*, whereas a rice homolog of these genes, *RSOsPR10*, was rapidly induced by a fungal infection, hypothetically under the jasmonic acid signaling pathway^53,61,62^. *DIR1* genes encode non-specific lipid transfer proteins which are required for the onset of systemic acquired resistance (SAR)^63–65^. During development of defense response, DIR1 protein is transported via phloem from the infection site to induce SAR in distant organs^65–67^. Interestingly, *DIR1* gene *TanjilG_02313* was significantly induced at the first time point in the 84A:476 and Population 22660 lines, however, anthracnose resistance was successfully developed only in the 84A:476 line. It may indicate some sub-functionalization of *DIR1* genes in NLL, as three other homologs were responsive to inoculation only in the 83A:476 line at 6 hpi and the direction of this response was downregulation.

### Contribution of redox components and ethylene signaling to anthracnose resistance

The most numerous components corresponding to biological process termed “GO:0055114 oxidation–reduction process” overrepresented in our study were cytochrome P450 proteins, peroxidases, linoleate 9S-/13S-lipoxygenases and 1-aminocyclopropane-1-carboxylate oxidases. Moreover, our WGCNA designated an *HSFA4a* homolog as a hub for a module carrying, among others, a candidate *Lanr1* resistance gene, *TanjilG_05042*. *HSFA4a* is a component of redox-dependent regulation of nuclear transcription in plants^68^.

Cytochrome P450 proteins are oxidoreductases that catalyze NADPH- and/or O2-dependent hydroxylation reactions in primary and secondary metabolism, including xenobiotic metabolism as well as biosynthesis of hormones, fatty acids, sterols, cell wall components, biopolymers, and defense compounds^69^. Variability of functions performed by cytochrome P450s in plants has been reflected in our study by the high number of homologs with altered expression (37) and by differences in the type of response between particular genes from − 10.6 log2(fold-change) downregulation to 5.7 upregulation. Elucidation of hypothetical biological function of NLL genes in such a large protein superfamily using only RNA-seq data would be very speculative. Nevertheless, it is worth mentioning that some cytochrome P450 genes were associated with increased resistance to pathogenic fungi or bacteria, including contribution to hypersensitive response^69–71^.

Class III peroxidases are multifunctional plant enzymes involved in a broad range of metabolic processes throughout plant growth and development as well as in the response to environmental stress, such as salinity, drought, high light intensity and pathogen attack^72^. Peroxidases were involved in interaction of several plant species with *Colletotrichum* spp., including *Stylosanthes humilis* and *C. gloeosporioides*, *Lens culinaris* and *C. truncatum*, *Phaseolus vulgaris* and *C. lindemuthianum*, *Cucumis sativus* and *C. lagenarium*^73–76^. Response was very quick, sometimes even at 4 hpi, preceding fungal penetration of plant tissues^73^. Peroxidase genes were also responsive to the inoculation of NLL with *D. toxica*^53^. Besides typical function in regulation of oxidative burst or elimination of oxidative stress, peroxidases may also hamper the pathogen growth by constituting a physical barrier based on cell wall reinforcement in the processes of lignification, suberization or cross-linking of particular compounds^77^. Such a function can be in silico attributed to a *TanjilG_03329* gene encoding putative lignin-forming anionic peroxidase, which was significantly upregulated in our study at 6 hpi in the resistant line 83A:476 and non-responsive in other lines and time points.

Linoleate 9S-/13S-lipoxygenases commit the first step in oxylipin biosynthesis pathway^78^. Products of this pathway play diversified functions in plant defense, involving strengthening of the cell wall by formation of callose and pectin deposits, as well as modulation of oxidative stress by the production of reactive oxygen species^79–83^. In the present study expression of linoleate 9S-/13S-lipoxygenase was altered in all lines but at different time points with the prevalence of upregulation in the susceptible Population 22660 and downregulation in lines carrying resistant *Lanr1* and *AnMan* alleles, highlighting diversification of oxylipin tier of anthracnose defense response between these genotypes.

1-aminocyclopropane-1-carboxylate oxidase (*ACO*) homologs were significantly upregulated (9 genes) or downregulated (2 genes) in reaction to *C. lupini* inoculation. With just two exceptions, all these responses occurred at 6 hpi in 83A:476. Enzymatic reaction conferred by the ACO protein is the rate-limiting step in ethylene production and as such is under stringent regulation^84^. Ethylene is a phytohormone with multiple roles in regulation of developmental processes as well as plant responses to abiotic and biotic stress conditions^85^. Induction of *ACO* transcription and activation of ethylene signaling pathways is associated with improved rice resistance to the hemibiotrophic fungus *Magnaporthe oryzae* by regulating reactive oxygen species and phytoalexin production^86,87^. High similarity in leaf infection process found between *M. oryzae* and *C. lupini*^88,89^, confronted with a huge upregulation of *ACO* homologs in the 83A:476 line reported in this study, moves ethylene signaling center stage of possible molecular pathways conferring NLL anthracnose resistance.

### Downregulation of photosynthesis-related genes

In the present study, large-scale downregulation of many photosynthesis-related genes was observed at 6 hpi in 83A:476, as well as at 48 hpi in Mandelup and Population 22660. The range and earliness of these changes were proportional to the level of anthracnose resistance observed in this experiment. The strong and early suppression of photosynthesis-related transcripts has been recently reported for several plant–pathogen interaction models, including pathogenic bacteria and fungi^90–93^. Hasty (since 2 hpi in some interactions) and global downregulation of photosynthesis-related genes in response to infection may trigger plant immunity based on deployment of reactive oxygen species and their interactions with salicylic acid pathway in mediation of hypersensitive response^90,94^.

### Proposed mechanism of resistance

To summarize, proposed mechanism of defense response in the most resistant line (83A:476) includes rapid recognition of the pathogen by R gene (putatively TIR-NBS-LRR *TanjilG_05042*) and hypersensitive response mediated by salicylic acid and ethylene signaling pathways, followed by establishment of long distance SAR maintained by DIR-1 proteins. It should be noted that biotrophic phase during lupin *C. lupini* infection is surprisingly short (it takes about 2 days), and is followed by the necrotrophic growth afterwards^95^. The switch between these phases may be associated with the expression of necrosis- and ethylene-inducing proteins, which act as elicitors of the hypersensitive response in a plant host^96^. Therefore, time window available for successful arrest of *C. lupini* at the biotrophic phase is very narrow. Reprogramming of redox- and photosynthesis-related genes observed at 6 hpi in 83A:476 stays in line with the progress of fungal hyphae and prognosticates development of successful defense response at biotrophic phase. Transcriptomic response of Mandelup and Population 22660 is probably too delayed to trap the fungus before the switch to necrotrophic growth, however, Mandelup may be more efficient than Population 22660 by the relatively quick regulation of PR-10 proteins contributing to the horizontal resistance.

ETI driven by classic R genes seems to be common anthracnose resistance mechanism in legumes. Thus, in the model legume species, *Medicago truncatula*, resistance to anthracnose is conferred by the *RCT1* gene, which is a member of TIR-NBS-LRR class of plant R genes^97^. This gene conferred also broad-spectrum anthracnose resistance in alfalfa, when transferred into susceptible plants^98^. In common bean, (*P. vulgaris*), more than twenty anthracnose resistance genes have been identified hitherto. Some of these genes were found in regions lacking any typical R gene, however, many others were localized at the edge of chromosomes carrying clusters of NBS-LRR genes, including also TIR-NBS-LRRs^99^. Association of NBS-LRR genes with anthracnose resistance in common bean was also evidenced by genome-wide SSR study^100^. Classic R genes were also found in genome regions carrying major anthracnose resistance loci in white lupin^101^.

## Conclusions

Our work has demonstrated that an immediate resistance response activated at early stages of plant infection (preferably not later than 12 hpi) efficiently protected narrow-leafed lupin from anthracnose caused by the pathogenic fungus *Collelotrichum lupini*. Using high-throughput sequencing we have demonstrated differential gene expression profiles in NLL plants against anthracnose, conferred by *Lanr1* and *AnMan* resistance genes*.* A successful defense involved orchestrated reprogramming of oxidation–reduction, photosynthesis, and pathogenesis-related protein genes within a few hours after the first contact between the plant and the pathogen. Similar defense response but delayed in time, was much less efficient in plant protection against the disease. Anthracnose resistance driven by *Lanr1* gene resembles typical rapid R gene response (effector-triggered immunity) whereas *AnMan* gene confers most likely horizontal response (microbe-associated molecular pattern-triggered immunity) providing moderate level of resistance.

## Material and methods

### Plant material used in the study

The set of 215 NLL lines which was used for anthracnose marker screening consisted of 74 cultivars, 60 cross derivatives or breeding lines, 5 mutants and 76 wild or primitive accessions. These lines originated from 17 countries, predominantly from Poland (58 accessions), Spain (47 accessions), Germany (27 accessions), Australia (26 accessions), Russia (19 accessions), Belarus (7 accessions), Italy (5 accessions), and the remaining lines from 10 countries. This set included also reference resistant lines: 83A:476, Tanjil, Wonga carrying *Lanr1* allele and Mandelup carrying *AnMan* allele. Lines were derived from the European Lupin Gene Resources Database maintained by Poznań Plant Breeding Ltd. in Wiatrowo, Poland (Supplementary Table S1).

### Screening of anthracnose resistance markers

Plants were grown in controlled conditions (photoperiod 16 h, temperature 25 °C day and 18 °C night). Two biological replicates were analyzed. DNA was isolated from three-week old leaves with the use of DNeasy Plant Mini Kit (Qiagen, Hilden, Germany), according to the protocol. The quality and concentration of isolated DNA were evaluated by spectrophotometer method (NanoDrop 2000; Thermo Fisher Scientific, Waltham, MA, USA). Marker AnManM1 tagging *AnMan* anthracnose resistance gene (originating from the cv. Mandelup) as well as markers Anseq3 and Anseq4 flanking *Lanr1* gene (originating from the cv. Tanjil) were analyzed^11,26,28^. Resistant allele homozygotes were scored as “1”, susceptible as “0”, whereas heterozygotes as 0.5.

### Evaluation of anthracnose resistance in controlled conditions

Based on the results of AnManM1, AnSeq3 and AnSeq4 markers screening and seed availability for eventual downstream experiments, fifty NLL lines were selected for anthracnose resistance phenotyping. The assay was performed in a computer-controlled greenhouse under 14-h photoperiod and with a temperature regime of a 22 °C day vs 19 °C night in two independent replications. Seeds were scarified (by cutting seed coat with the sharp razor blade on the opposite side than the embryo) before sowing to stop seed dormancy resulting from hard seed coat and provide even germination. Pots (11 × 11 × 21 cm) with sterilized soil (TS-1 REC 085 Medium Basic, Klasmann-Deilmann Polska, Warsaw, Poland) were used for plant cultivation. The inoculation was done using *Colletotrichum lupini* strain Col-08, obtained in 1999 from the stem of narrow-leafed lupin plants cultivated in the field located in Wierzenica (52° 27′ 42″ N 17° 04′ 05″ E) in Great Poland region. The isolate was cultured on SNA medium for 21 days at 20 °C under black light to induce sporulation. The inoculation was performed 4 weeks after sowing, when the plants reached 4–6 leaf stage, by spraying of conidial spore suspension at the concentration 0.5 × 10^6^ conidia per ml. After inoculation, plants were kept for 24 h in darkness under ~ 98% humidity and the temperature 25 °C to facilitate conidia germination and infection process. Afterwards, plants were grown under 14-h photoperiod in temperature regime 22 °C day/19 °C night and 70% humidity. Disease scoring was performed 22 days post inoculation and was based on the presence of necrotic lesions on stems and leaves in the scale from 0 (immune) to 9 (extremely susceptible). Moreover, weight of plants was measured after scoring. Relation between marker genotypes and disease phenotypes was calculated as point-biserial correlation (there was no heterozygote marker score in the set of lines subjected to anthracnose resistance phenotyping).

### Experiment for gene expression profiling

Based on the results of anthracnose resistance phenotyping and *Lanr1*/*AnMan* marker genotyping, four NLL accessions were selected for gene expression profiling (Table 1). This set included cv. Mandelup (resistant allele *AnMan*), parental line of mapping population 83A:476 (resistant allele *Lanr1*), cv. Boregine (putative novel donor of resistance), and wild accession Population 22660 (susceptible). Plant cultivation conditions and inoculation pattern were the same as in the anthracnose resistance phenotyping experiment. Leaves were sampled at 6, 12, 24, 36, and 48 h post inoculation (hpi), both from studied (inoculated) and control (mock-inoculated) plants, immediately frozen in liquid nitrogen and stored at − 80 °C until RNA isolation. Disease scoring was performed 22 days post inoculation.

### RNA isolation

Frozen leaf tissue (50 mg) was homogenized in 2 ml tubes (Eppendorf, Hamburg, Germany) with two stainless steel beads (ø 5 mm) using TissueLyser II (Qiagen). SV Total RNA Isolation System (Promega, Madison, WI, USA) was used for RNA isolation without any changes to the protocol except extending DNAse I digestion to 25 min. RNA quality was measured using an Experion™ Automated Electrophoresis System (Bio-Rad, Hercules, CA, USA). and NanoDrop 2000 (Thermo Fisher Scientific) (Supplementary Table S3).

### RNA sequencing and data analysis

Samples at four time points (6 h, 12 h, 24 h and 48 h post-inoculation) with three biological replications were analyzed. RNA libraries were prepared (TruSeq RNA Sample Prep Kit v2, Illumina, San Diego, CA, USA) and sequenced (NovaSeq 6000, Illumina) exploiting the 100 bp paired-end protocol and 6 Gbp (60 M reads) expected sequencing coverage (Macrogen, Seoul, Republic of Korea). Removing of the adapter-related sequences and quality trimming were performed using AdapterRemoval ver 2.1.7^102^ (parameters: minquality 20, minlength 50). Mapping to the NLL reference sequence LupAngTanjil_v1.0 (EnsemblPlants) was done in TopHat ver. 2.1.1^103^ (parameters: no-mixed-library-type, fr-unstranded, -no-discordant, others at default values). Counting of reads aligned to annotated transcripts was performed using the function featureCounts in Bioconductor, R 3.5.1 (Rsubread library)^104^, and was followed by submission of the count data to differential expression analysis in Deseq2 in R^105^. Genes that were characterized by a base mean expression of at least 5, |log_2_(Fold Change)|> 2, and corrected P value < 0.05 were declared as differentially expressed in defined comparisons. An analysis of Gene Ontology terms enrichment was performed using the hypergeometric test, with computation of the family-wise error rates (FWER), using the GOfuncR library in Bioconductor^106^. Disease Resistance Analysis and Gene Orthology (DRAGO 2) tool in the Plant Resistance Genes database (PRGdb)^38^ was exploited to survey differentially expressed genes for the presence of typical R gene domains. A weighted gene co-expression network analysis was performed using the WGCNA package in R^107,108^ (parameters: beta = 6, average link clustering method, cutHeight = 0.90, minSize = 50).

### Quantitative gene expression profiling

The set of genes analyzed by quantitative PCR included glucan endo-1,3-beta-glucosidase-like (*TanjilG_23384*), LlR18A (*TanjilG_27015*), acidic endochitinase (*TanjilG_04706*), HSP17.4 (*TanjilG_05080*), a candidate gene for the *Lanr1* locus—disease resistance protein (TIR-NBS-LRR class) (*TanjilG_05042*), a candidate gene for the *AnMan* locus—a rho GTPase-activating protein (*TanjilG_12861*), and a legume-specific hypothetical protein significantly upregulated in the majority of line × time point combinations (*TanjilG_10657*). Reference genes validated in the previous NLL quantitative gene expression studies were selected, namely *LanDExH7* (*TanjilG_23733*) and *LanTUB6* (*TanjilG_32899*)^53,109–112^. Primers were designed in Geneious Prime (Auckland, New Zealand) using Primer3^113,114^. Standard curves were developed for all analyzed genes using the same method as in previous narrow-leafed lupin study^112^. R^2^ and PCR efficiency values were calculated in Bio-Rad CFX Manager 3.1 (Supplementary Table S9). First-strand cDNA was synthesized using GoScript(TM) Reverse Transcription System (Promega) and 5 μg of total RNA per sample. Quantitative PCR was performed using 96-well PCR plates (Eppendorf) with inter-run calibration samples (*LanTUB6*) and no template controls included on all plates. All reactions were run in 3 technical replications. Two-step PCR protocol was exploited using iTaq Universal SYBR Green Supermix (Bio-Rad) and CFX Connect Real-Time PCR Detection System (Bio-Rad). To control the specificity of amplification, high resolution melting (65–85 °C) was performed after every PCR. Calculations of ∆∆Cq were performed in Bio-Rad CFX Manager 3.1 taking into consideration PCR efficiency values and results obtained for both reference genes. Final computations (mean value and standard deviation) and visualization (graphs) were performed in Microsoft Excel 2010. Calculations were performed to check the response to inoculation (i.e., expression in the inoculated samples divided by expression in the control). Statistical significance was evaluated using t-test for mean ratio^115,116^ in R^117^ with a custom script using ‘t.test.ratio’ function from the mratios package. In the first step, the equality of variance was tested; if this condition was satisfied, the classical t-test formula was used; otherwise the Welch's t-test formula was used^118^.

### Ethical statement

Experimental research and field studies on plants (either cultivated or wild), including the collection of plant material, complies with relevant institutional, national, and international guidelines and legislation.

## Supplementary Information

Supplementary Information 1.

Supplementary Information 2.

Supplementary Information 3.

## Data Availability

All data generated during this study are included in this published article, its Supplementary Information files and in public repository (ArrayExpress database under accession number E-MTAB-11164).

## References

[1] Mousavi-Derazmahalleh M (2018). The western Mediterranean region provided the founder population of domesticated narrow-leafed lupin. Theor. Appl. Genet..

[2] Mousavi-Derazmahalleh M (2018). Exploring the genetic and adaptive diversity of a pan-Mediterranean crop wild relative: narrow-leafed lupin. Theor. Appl. Genet..

[3] Sengbusch R (1947). V 2.0 Jahre Süßlupinenforschung und Züchtung in Deutschland. Forschungen Fortschritte.

[4] von Sengbusch R (1942). Süßlupinen und Öllupinen. Die Entstehungsgeschichte einiger neuer Kulturpflanzen. Landwirtschaftliche Jahrbücher.

[5] Gladstones J, Hill G (1969). Selection for economic characters in *Lupinus angustifolius* and *L. digitatus*. 2. Time of flowering. Aust. J. Exp. Agric..

[6] Gladstones J (1967). Selection for economic characters in *Lupinus angustifolius* and *L. digitatus*. Aust. J. Exp. Agric..

[7] Mikołajczyk J (1966). Genetic studies in *Lupinus angustifolius*. 2. Inheritance of some morphological characters in blue lupine. Genet. Polon..

[8] Mikołajczyk J (1966). Genetic studies in *Lupinus angustifolius*. Part III. Inheritance of the alkaloid content, seed hardness and length of the growing season in blue lupin. Genet. Polon..

[9] Święcicki W, Święcicki WK (1995). Domestication and breeding improvement of narrow-leafed lupin (*L. angustifolius* L.). J. Appl. Genet..

[10] Stefanova KT, Buirchell B (2010). Multiplicative mixed models for genetic gain assessment in lupin breeding. Crop Sci..

[11] Plewiński P (2020). Innovative transcriptome-based genotyping highlights environmentally responsive genes for phenology, growth and yield in a non-model grain legume. Plant Cell Environ..

[12] Cowling WA, Karam BS, Lars GK, Matthew NN (2020). Genetic diversity in narrow-leafed lupin breeding after the domestication bottleneck. The Lupin Genome.

[13] Nirenberg HI, Feiler U, Hagedorn G (2002). Description of *Colletotrichum lupini* comb. nov. in modern terms. Mycologia.

[14] Weimer JL (1943). Anthracnose of lupines. Phytopathology.

[15] Weimer JL (1952). Lupine Anthracnose. Circular No 904.

[16] Forbes IJ, Wells HD (1961). Inheritance of resistance to anthracnose in blue lupines, *Lupinus angustifolius* L.. Crop Sci..

[17] Wells DH, Forbes I (1967). Effects of temperature on growth of *Glomerella cingulata* in vitro and on its pathogenicity to *L. angustifolius* genotypes. Phytopathology.

[18] Shea G, Palta JA, Berger JB (2008). 12th International Lupin Conference “Lupins for Health and Wealth”.

[19] Talhinhas P, Baroncelli R, Floch GL (2016). Anthracnose of lupins caused by *Colletotrichum lupini*: A recent disease and a successful worldwide pathogen. J. Plant Pathol..

[20] Gondran J, Hill GD (1996). Towards the 21st Century. Proceedings of the 8th International Lupin Conference.

[21] Gondran J (1984). Les maladies du lupin blanc doux en France. Perspect. Agricoles.

[22] Sweetingham M, Cowling WA, Buirchell B, Brown A, Shivas R (1995). Anthracnose of lupins in Western Australia. Australas. Plant Path..

[23] Frencel, I. M., Lewartowska, E. & Czerwińska, A. In *4th International Symposium of the European Foundation for Plant Pathology.* (eds H. W. Dehne *et al.*) 303–306 (Springer Netherlands, 1997).

[24] Frencel IM (1998). Report on first detection of anthracnose (*Colletotrichum gloeosporioides*) on lupins in Poland. Plant Dis..

[25] Yang H, Boersma JG, You M, Buirchell BJ, Sweetingham MW (2004). Development and implementation of a sequence-specific PCR marker linked to a gene conferring resistance to anthracnose disease in narrow-leafed lupin (*Lupinus angustifolius* L.). Mol. Breed..

[26] Yang H, Renshaw D, Thomas G, Buirchell B, Sweetingham M (2008). A strategy to develop molecular markers applicable to a wide range of crosses for marker assisted selection in plant breeding: a case study on anthracnose disease resistance in lupin (*Lupinus angustifolius* L.). Mol. Breed..

[27] You M (2005). A PCR-based molecular marker applicable for marker-assisted selection for anthracnose disease resistance in lupin breeding. Cell. Mol. Biol. Lett..

[28] Yang H (2012). Application of next-generation sequencing for rapid marker development in molecular plant breeding: a case study on anthracnose disease resistance in *Lupinus angustifolius* L.. BMC Genom..

[29] Yang H (2013). Draft genome sequence, and a sequence-defined genetic linkage map of the legume crop species *Lupinus angustifolius* L.. PLoS One.

[30] Yang H (2015). Sequencing consolidates molecular markers with plant breeding practice. Theor. Appl. Genet..

[31] Boersma JG (2005). Construction of a genetic linkage map using MFLP and identification of molecular markers linked to domestication genes in narrow-leafed lupin (*Lupinus angustifolius* L.). Cell. Mol. Biol. Lett..

[32] Kamphuis LG (2015). Transcriptome sequencing of different narrow-leafed lupin tissue types provides a comprehensive uni-gene assembly and extensive gene-based molecular markers. Plant Biotechnol. J..

[33] Nelson MN (2006). The first gene-based map of *Lupinus angustifolius* L.-location of domestication genes and conserved synteny with *Medicago truncatula*. Theor. Appl. Genet..

[34] Zhou G (2018). Construction of an ultra-high density consensus genetic map, and enhancement of the physical map from genome sequencing in *Lupinus angustifolius*. Theor. Appl. Genet..

[35] Hane JK (2017). A comprehensive draft genome sequence for lupin (*Lupinus angustifolius*), an emerging health food: insights into plant-microbe interactions and legume evolution. Plant Biotechnol. J..

[36] Fischer K (2015). Characterization and mapping of LanrBo: a locus conferring anthracnose resistance in narrow-leafed lupin (*Lupinus angustifolius* L.). Theor. Appl. Genet..

[37] Aslam MM (2020). In vitro regeneration potential of white lupin (*Lupinus albus*) from cotyledonary nodes. Plants (Basel).

[38] Osuna-Cruz CM (2018). PRGdb 3.0: a comprehensive platform for prediction and analysis of plant disease resistance genes. Nucleic Acids Res..

[39] Cowling WA (1999). Pedigrees and characteristics of narrow-leafed lupin cultivars released in Australia from 1967 to 1998. Bull. Agric. Western Aust..

[40] Grishin SY (2015). Identification of the Lanr1 gene of resistance to anthracnose of narrow-leafed lupine (*Lupinus angustifolius* L.) using DNA-markers AnSeq3 and AnSeq4. Sel'skokhozyaistvennaya Biol..

[41] Sweetingham, M. W. *et al.* in *México, where old and new world lupins meet. 11th International Lupin Conference.* (eds E. van Santen & G.D. Hill) 2–5 (International Lupin Association, 2006).

[42] Thomas GJ, Sweetingham MW, Yang HA, Speijers J (2008). Effect of temperature on growth of *Colletotrichum lupini* and on anthracnose infection and resistance in lupins. Australas. Plant Path..

[43] Książkiewicz M, Yang H, Singh KB, Kamphuis LG, Nelson MN (2020). Molecular marker resources supporting the Australian lupin breeding program. The Lupin Genome.

[44] Staples RC, Hoch HC (1987). Infection structures—Form and function. Exp. Mycol..

[45] Nesher I, Barhoom S, Sharon A (2008). Cell cycle and cell death are not necessary for appressorium formation and plant infection in the fungal plant pathogen *Colletotrichum gloeosporioides*. BMC Biol..

[46] Hwang CS, Flaishman MA, Kolattukudy PE (1995). Cloning of a gene expressed during appressorium formation by *Colletotrichum gloeosporioides* and a marked decrease in virulence by disruption of this gene. Plant Cell.

[47] Buhr TL, Dickman MB (1997). Gene expression analysis during conidial germ tube and appressorium development in *Colletotrichum trifolii*. Appl. Environ. Microbiol..

[48] Byrne JM, Hausbeck MK, Hammerschmidt R (1997). Conidial germination and appressorium formation of *Colletotrichum coccodes* on tomato foliage. Plant Dis..

[49] Arroyo FT (2002). Development of *Colletotrichum acutatum* in the foliar tissue of strawberry plants. Plant Protect. Sci..

[50] Jones JDG, Dangl JL (2006). The plant immune system. Nature.

[51] Newman M-A, Sundelin T, Nielsen J, Erbs G (2013). MAMP (microbe-associated molecular pattern) triggered immunity in plants. Front. Plant Sci..

[52] Balint-Kurti P (2019). The plant hypersensitive response: concepts, control and consequences. Mol. Plant Pathol..

[53] Książkiewicz M (2021). The resistance of narrow-leafed lupin to *Diaporthe toxica* is based on the rapid activation of defense response genes. Int. J. Mol. Sci..

[54] Crowell DN, John ME, Russell D, Amasino RM (1992). Characterization of a stress-induced, developmentally regulated gene family from soybean. Plant Mol. Biol..

[55] Fujita K, Inui H (2021). Review: Biological functions of major latex-like proteins in plants. Plant Sci..

[56] Chen JY, Dai XF (2010). Cloning and characterization of the *Gossypium hirsutum* major latex protein gene and functional analysis in *Arabidopsis thaliana*. Planta.

[57] Zhu L (2017). Transcriptomics analysis of apple leaves in response to *Alternaria alternata* apple pathotype infection. Front. Plant Sci..

[58] Pérez-Torres C-A (2021). Molecular evidence of the avocado defense response to *Fusarium kuroshium* infection: a deep transcriptome analysis using RNA-Seq. PeerJ.

[59] He S (2020). Major latex protein MdMLP423 negatively regulates defense against fungal infections in apple. Int. J. Mol. Sci..

[60] Leitão ST, Santos C, Araújo SDS, Rubiales D, Vaz Patto MC (2021). Shared and tailored common bean transcriptomic responses to combined fusarium wilt and water deficit. Hortic. Res..

[61] Sikorski MM (1999). Expression of genes encoding PR10 class pathogenesis-related proteins is inhibited in yellow lupine root nodules. Plant Sci..

[62] Hashimoto M (2004). A novel rice PR10 protein, RSOsPR10, specifically induced in roots by biotic and abiotic stresses, possibly via the jasmonic acid signaling pathway. Plant Cell Physiol..

[63] Lascombe MB (2008). The structure of "defective in induced resistance" protein of *Arabidopsis thaliana*, DIR1, reveals a new type of lipid transfer protein. Protein Sci..

[64] Maldonado AM, Doerner P, Dixon RA, Lamb CJ, Cameron RK (2002). A putative lipid transfer protein involved in systemic resistance signalling in *Arabidopsis*. Nature.

[65] Carella P, Kempthorne CJ, Wilson DC, Isaacs M, Cameron RK (2017). Exploring the role of DIR1, DIR1-like and other lipid transfer proteins during systemic immunity in *Arabidopsis*. Physiol. Mol. Plant Pathol..

[66] Cameron RK (2016). Using DIR1 to investigate long-distance signal movement during systemic acquired resistance. Can. J. Plant Pathol..

[67] Champigny MJ (2013). Long distance movement of DIR1 and investigation of the role of DIR1-like during systemic acquired resistance in *Arabidopsis*. Front Plant Sci.

[68] He H, Van Breusegem F, Mhamdi A (2018). Redox-dependent control of nuclear transcription in plants. J. Exp. Bot..

[69] Pandian BA, Sathishraj R, Djanaguiraman M, Prasad PVV, Jugulam M (2020). Role of cytochrome P450 enzymes in plant stress response. Antioxidants (Basel).

[70] Yan Q (2016). *GmCYP82A3*, a soybean cytochrome P450 family gene involved in the jasmonic acid and ethylene signaling pathway, enhances plant resistance to biotic and abiotic stresses. PLoS One.

[71] Kong L, Anderson JM, Ohm HW (2005). Induction of wheat defense and stress-related genes in response to *Fusarium graminearum*. Genome.

[72] Veljović Jovanović S, Kukavica B, Vidović M, Morina F, Menckhoff L, Gupta DK, Palma JM, Corpa FJ (2018). Class III peroxidases: functions, localization and redox regulation of isoenzymes. Antioxidants and Antioxidant Enzymes in Higher Plants.

[73] Harrison SJ, Curtis MD, McIntyre CL, Maclean DJ, Manners JM (1995). Differential expression of peroxidase isogenes during the early stages of infection of the tropical forage legume *Stylosanthes humilis* by *Colletotrichum gloeosporioides*. Mol. Plant-Microbe Interact..

[74] Bhadauria V (2013). Identification of *Lens culinaris* defense genes responsive to the anthracnose pathogen *Colletotrichum truncatum*. BMC Genet..

[75] Ombiri J, Zinkernagel V, Gathuru EM, Achwanya O (2002). Induction of ethylene biosynthesis and peroxidase activity in bean genotypes inoculated with *Colletotrichum lindemuthianum*, and their role as indicators of resistance or susceptibility. J. Plant Dis. Prot..

[76] Hammerschmidt R, Nuckles EM, Kuć J (1982). Association of enhanced peroxidase activity with induced systemic resistance of cucumber to *Colletotrichum lagenarium*. Physiol. Plant Pathol..

[77] Almagro L (2008). Class III peroxidases in plant defence reactions. J. Exp. Bot..

[78] Wasternack C (2007). Jasmonates: an update on biosynthesis, signal transduction and action in plant stress response, growth and development. Ann. Bot..

[79] Vicente J (2012). Role of 9-lipoxygenase and α-dioxygenase oxylipin pathways as modulators of local and systemic defense. Mol. Plant.

[80] Blée E (2002). Impact of phyto-oxylipins in plant defense. Trends Plant Sci..

[81] Genva M (2019). New insights into the biosynthesis of esterified oxylipins and their involvement in plant defense and developmental mechanisms. Phytochem. Rev..

[82] López MA (2011). Antagonistic role of 9-lipoxygenase-derived oxylipins and ethylene in the control of oxidative stress, lipid peroxidation and plant defence. Plant J..

[83] Vellosillo T (2007). Oxylipins produced by the 9-lipoxygenase pathway in *Arabidopsis* regulate lateral root development and defense responses through a specific signaling cascade. Plant Cell.

[84] Houben M, Van de Poel B (2019). 1-Aminocyclopropane-1-carboxylic acid oxidase (ACO): The enzyme that makes the plant hormone ethylene. Front. Plant Sci..

[85] Khan NA, Khan MIR, Ferrante A, Poor P (2017). Editorial: ethylene: a key regulatory molecule in plants. Front. Plant Sci..

[86] Yang C (2017). Activation of ethylene signaling pathways enhances disease resistance by regulating ROS and phytoalexin production in rice. Plant J..

[87] Iwai T, Miyasaka A, Seo S, Ohashi Y (2006). Contribution of ethylene biosynthesis for resistance to blast fungus infection in young rice plants. Plant Physiol..

[88] Dubrulle G (2020). Deciphering the infectious process of *Colletotrichum lupini* in lupin through transcriptomic and proteomic analysis. Microorganisms.

[89] Wilson RA, Talbot NJ (2009). Under pressure: investigating the biology of plant infection by *Magnaporthe oryzae*. Nat. Rev. Microbiol..

[90] Yang H, Luo P (2021). Changes in photosynthesis could provide important insight into the interaction between wheat and fungal pathogens. Int. J. Mol. Sci..

[91] Göhre V, Jones AM, Sklenář J, Robatzek S, Weber AP (2012). Molecular crosstalk between PAMP-triggered immunity and photosynthesis. Mol. Plant-Microbe Interact..

[92] de Torres Zabala M (2015). Chloroplasts play a central role in plant defence and are targeted by pathogen effectors. Nature plants.

[93] Swarbrick PJ, Schulze-Lefert P, Scholes JD (2006). Metabolic consequences of susceptibility and resistance (race-specific and broad-spectrum) in barley leaves challenged with powdery mildew. Plant Cell Environ.

[94] Hu Y (2020). Potential role of photosynthesis in the regulation of reactive oxygen species and defence responses to *Blumeria graminis* f. sp. tritici in wheat. Int. J. Mol. Sci..

[95] Dubrulle G (2020). Phylogenetic diversity and effect of temperature on pathogenicity of *Colletotrichum lupini*. Plant Dis..

[96] Pemberton CL, Salmond GPC (2004). The Nep1-like proteins—a growing family of microbial elicitors of plant necrosis. Mol. Plant Pathol..

[97] Yang S (2007). Genetic and physical localization of an anthracnose resistance gene in *Medicago truncatula*. Theor. Appl. Genet..

[98] Yang S (2008). Alfalfa benefits from *Medicago truncatula*: the RCT1 gene from M truncatula confers broad-spectrum resistance to anthracnose in alfalfa. Proc. Natl. Acad. Sci. U S A.

[99] Meziadi C (2016). Development of molecular markers linked to disease resistance genes in common bean based on whole genome sequence. Plant Sci..

[100] Wu J, Zhu J, Wang L, Wang S (2017). Genome-wide association study identifies NBS-LRR-encoding genes related with anthracnose and common bacterial blight in the common bean. Front. Plant Sci..

[101] Rychel-Bielska S (2020). Development of PCR-based markers and whole-genome selection model for anthracnose resistance in white lupin (*Lupinus albus* L.). J. Appl. Genet..

[102] Schubert M, Lindgreen S, Orlando L (2016). AdapterRemoval v2: rapid adapter trimming, identification, and read merging. BMC. Res. Notes.

[103] Kim D (2013). TopHat2: accurate alignment of transcriptomes in the presence of insertions, deletions and gene fusions. Genome Biol..

[104] Liao Y, Smyth GK, Shi W (2019). The R package Rsubread is easier, faster, cheaper and better for alignment and quantification of RNA sequencing reads. Nucleic Acids Res..

[105] Love MI, Huber W, Anders S (2014). Moderated estimation of fold change and dispersion for RNA-seq data with DESeq2. Genome Biol..

[106] GOfuncR: Gene ontology enrichment using FUNC. R package version 1.10.0 (2020).

[107] Langfelder P, Horvath S (2012). Fast R functions for robust correlations and hierarchical clustering. J. Stat. Softw..

[108] Langfelder P, Horvath S (2008). WGCNA: an R package for weighted correlation network analysis. BMC Bioinform..

[109] Taylor CM, Jost R, Erskine W, Nelson MN (2016). Identifying stable reference genes for qRT-PCR normalisation in gene expression studies of narrow-leafed lupin (*Lupinus angustifolius* L.). PLoS One.

[110] Taylor CM (2019). INDEL variation in the regulatory region of the major flowering time gene LanFTc1 is associated with vernalization response and flowering time in narrow-leafed lupin (*Lupinus angustifolius* L.). Plant Cell Environ..

[111] Nelson MN (2017). The loss of vernalization requirement in narrow-leafed lupin is associated with a deletion in the promoter and de-repressed expression of a *Flowering Locus T* (*FT*) homologue. New Phytol..

[112] Rychel-Bielska S, Plewiński P, Kozak B, Galek R, Książkiewicz M (2020). Photoperiod and vernalization control of flowering-related genes: a case study of the narrow-leafed lupin (*Lupinus angustifolius* L.). Front. Plant Sci..

[113] Kearse M (2012). Geneious Basic: an integrated and extendable desktop software platform for the organization and analysis of sequence data. Bioinformatics.

[114] Untergasser A (2012). Primer3—new capabilities and interfaces. Nucleic Acids Res..

[115] Hauschke D, Kieser M, Hothorn LA (1999). Proof of safety in toxicology based on the ratio of two means for normally distributed data. Biom. J..

[116] Tamhane AC, Logan BR (2004). Finding the maximum safe dose level for heteroscedastic data. J. Biopharm. Stat..

[117] R Core Team. (Vienna, Austria, 2013).

[118] Welch BL (1947). The generalization of student's' problem when several different population variances are involved. Biometrika.

