# Supplementary material for: A successful defense of the narrow-leafed lupin against anthracnose involves quick and orchestrated reprogramming of oxidation–reduction, photosynthesis and pathogenesis-related genes
Source: Sci Rep. 2022 May 17;12:8164. doi: 10.1038/s41598-022-12257-7 (PMC9114385; doi:10.1038/s41598-022-12257-7)
Supplement: Supplementary file 1 — Supplementary Information 1. [file 41598_2022_12257_MOESM1_ESM.pdf]

Michał Książkiewicz, Sandra Rychel-Bielska, Piotr Plewiński, Wojciech Bielski, Maria Nuc, Bartosz Kozak, Paweł Krajewski and Małgorzata Jędryczka

A successful defense of the narrow-leafed lupin against anthracnose involves quick and orchestrated reprogramming of oxidation-reduction, photosynthesis and pathogenesis-related genes

Scientific Reports

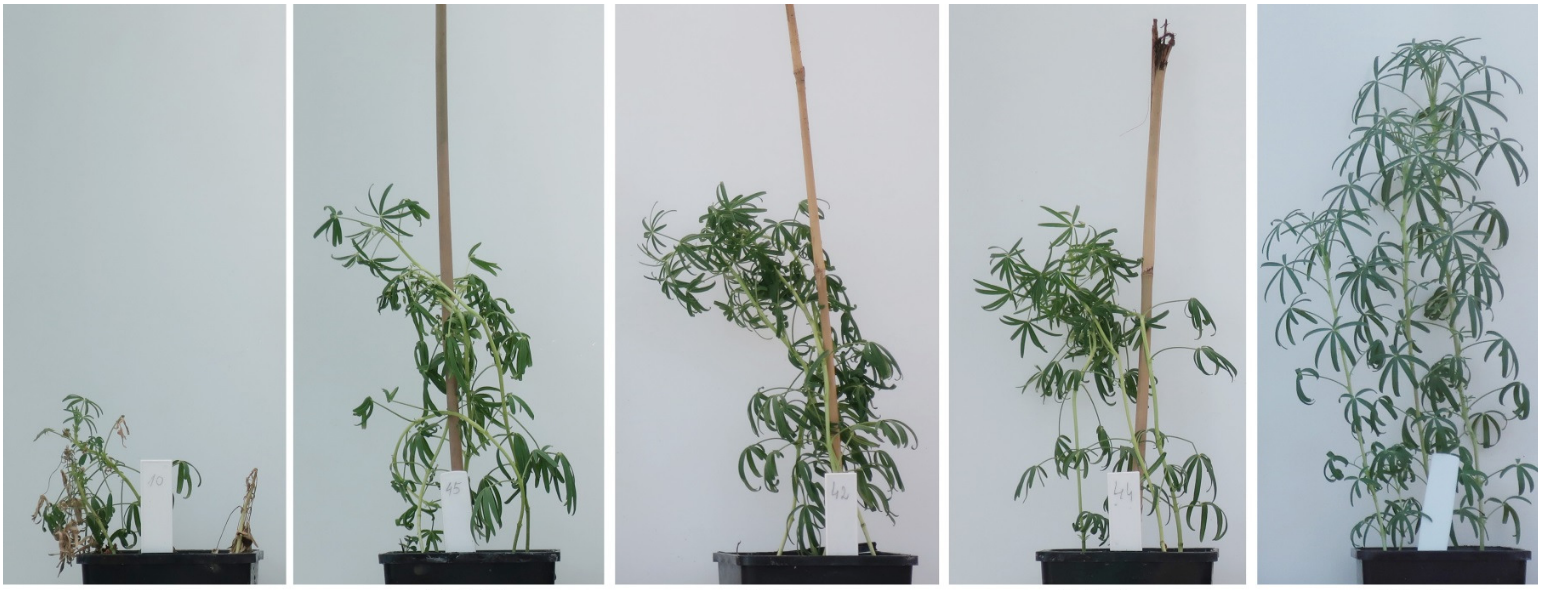

95843 Population 22660 (inoculated)	962351 Boregine (inoculated)	96230 Mandelup (inoculated)	96233 83A:476 (inoculated)	95843 Population 22660 (control)

Supplementary Figure 1. Comparison of plant growth at 22nd day after inoculation of selected narrow-leafed lupin lines with *Colletotrichum lupini*. Strain Col-08, obtained in 1999 from the stem of narrow-leafed lupin plants cultivated in the field located in Wierzenica (52°27′42″N 17°04′05″E), was used for inoculation. The inoculation was performed 4 weeks after sowing, when the plants reached 4-6 leaf stage, by spraying of conidial spore suspension at the concentration 0.5×10^6 conidia per ml. After inoculation, plants were kept for 24 hours in darkness under ~98% humidity and the temperature 25°C to facilitate conidia germination and infection process. Afterwards, plants were grown under 14-h photoperiod in temperature regime 22°C day / 19°C night and 70% humidity. The set of plants includes a wild accession Population 22660 (susceptible), cultivar Boregine (carrying a putative novel donor of resistance), cultivar Mandelup (carrying a resistant allele *AnMan*), parental line of mapping population 83A:476 (carrying resistant allele *Lanr1*). Non-inoculated control (shown on the right panel) was cultivated in the same conditions for the same period.
