# Supplementary material for: A successful defense of the narrow-leafed lupin against anthracnose involves quick and orchestrated reprogramming of oxidation–reduction, photosynthesis and pathogenesis-related genes
Source: Sci Rep. 2022 May 17;12:8164. doi: 10.1038/s41598-022-12257-7 (PMC9114385; doi:10.1038/s41598-022-12257-7)
Supplement: Supplementary file 3 — Supplementary Information 3. [file 41598_2022_12257_MOESM3_ESM.docx]

**Supplementary Material**

Supplementary Table S1. List of narrow-leafed lupin lines used in the study and results of anthracnose marker screening.

Supplementary Table S2. Supplementary Table S2. Results of the Lanr1 (Anseq3 and Anseq4) and AnMan (AnManM1) marker screening and controlled environment anthracnose resistance phenotyping of selected narrow-leafed lupin lines.

Supplementary Table S3. RNA concentration, RNA quality parameters and number of read pairs obtained from RNA sequencing.

Supplementary Table S4. List of genes analyzed by RNA-seq profiling of narrow-leafed lupin response to inoculation with *Colletotrichum lupini*.

Supplementary Table S5 Log2(fold-change) values between inoculated and control narrow-leafed lupin plants.

Supplementary Table S6. Genes significantly downregulated (-1) or upregulated (1) at at least one time point x line combination.

Supplementary Table S7. Typical R gene domains found in genes upregulated at 6 hours post inoculation.

Supplementary Table S8. Overrepresented gene ontology terms identified for the sets of differentially expressed genes.

Supplementary Table S9. Designed primers and calculated PCR efficiency and R-squared values for quantitative PCR.

Supplementary Table S10. Results of gene expression profiling by quantitative PCR.

Supplementary Table S11. Comparison of gene expression levels between inoculated and control plants and statistical significance of observed differences.

Supplementary Table S12. Results of weighted gene co-expression network analysis.

Supplementary Table S13. Overrepresented Gene Ontology terms in modules identified by weighted gene co-expression network analysis.

Supplementary Figure 1. Comparison of plant growth at 22nd day after inoculation of selected narrow-leafed lupin lines with *Colletotrichum lupini*.
